# Biomechanically Informed Image Registration for Patient-Specific Aortic Valve Strain Analysis

**DOI:** 10.1007/s10439-026-04245-z

**Published:** 2026-06-23

**Authors:** Mohsen Nakhaei, Alison M. Pouch, Silvani Amin, Matthew Daemer, Christian Herz, Natalie Yushkevich, Lourdes Al Ghofaily, Nimesh Desai, Joseph Bavaria, Matthew A. Jolley, Wensi Wu

**Affiliations:** 1 Department of Anesthesiology and Critical Care Medicine, Children’s Hospital of Philadelphia, Philadelphia, PA, USA; 2 Department of Radiology and Bioengineering, University of Pennsylvania, Philadelphia, PA, USA; 3 Department of Anesthesiology, University of Pennsylvania, Philadelphia, PA, USA; 4 Department of Surgery, University of Pennsylvania, Philadelphia, PA, USA; 5 Department of Cardiac Surgery, Jefferson Health, Philadelphia, PA, USA; 6 Department of Mechanical Engineering and Applied Mechanics, University of Pennsylvania, Philadelphia, PA, USA; 7 Cardiovascular Institute, Children’s Hospital of Philadelphia, Philadelphia, PA, USA

**Keywords:** Aortic valve biomechanics, Finite element simulation, Image registration, Computational biomechanics

## Abstract

**Purpose:**

Aortic valve (AV) biomechanics play a critical role in maintaining normal cardiac function. Pathological variations, particularly in bicuspid valves, alter leaflet loading, increase strain, and accelerate disease progression. Accurate patient-specific characterization of valve geometry and deformation is essential, but existing imaging and computational methods often fail to capture rapid valve motion, discontinuous deformation and complex patient-specific features, limiting precise biomechanical assessment.

**Methods:**

To address these limitations, we developed an image registration framework coupled with the finite element method (FEM) to improve AV tracking and biomechanical evaluation. Patient-specific valve geometries from 4D echocardiography and CT were used to simulate AV closure and generate intermediate deformation states. These FEM-generated states facilitated leaflet tracking, while image registration corrected misalignment between simulations and imaging data.

**Results:**

In 20 patients, FEM-augmented registration improved tracking accuracy by 40% compared with direct registration. This improvement enabled bounded-uncertainty strain estimation by aligning leaflet geometry with patient imaging, partially compensating for uncertainties in boundary conditions and material assumptions. Using the improved tracking results, areal, Green-Lagrange, and deviatoric strains were quantified in adult trileaflet and bicuspid valves, as well as pediatric patients. Exploratory comparisons across valve groups suggest that age- and size-related differences in total strain between adult trileaflet and pediatric valves may be driven primarily by volumetric rather than deviatoric components.

**Conclusion:**

This FEM-augmented registration framework improves geometric tracking of the aortic valve and yields bounded-uncertainty leaflet strain estimates with potential to inform patient-specific AV deformation for individualized intervention planning and generation of complementary training data for learning-based methods.

## Introduction

The aortic valve (AV) plays an essential role in cardiovascular function by regulating blood flow from the left ventricle to the aorta and preventing regurgitation during diastole. In most healthy individuals, the trileaflet aortic valve (TAV) consists of three leaflets that undergo large, rapid, and highly anisotropic deformations throughout each cardiac cycle. These deformations expose valvular interstitial cells (VICs) to cyclic strains that regulate extracellular matrix (ECM) and maintain tissue homeostasis [1-4]. Strain thus serves as the critical mechanobiological link connecting valve structure and the hemodynamic forces imposed on the valve to cellular behavior and long-term tissue remodeling. When strain patterns become abnormal (e.g., due to age-related degeneration, altered hemodynamics, or congenital defects), VICs activation and ECM dysregulation accelerate disease progression, as seen in calcific aortic valve disease (CAVD) [3-9].

Among congenital defects, bicuspid aortic valve (BAV) provides a clear example of how altered strain drives pathology. BAV, affecting 1–2% of the population [10], results from fusion of adjacent leaflets, producing asymmetric geometry and disordered loading. These structural changes elevate localized stresses and strains, particularly near the leaflet raphe [11-13]. These mechanical abnormalities contribute to early ECM disorganization, rapid calcification, and accelerated onset of aortic stenosis (AS) or regurgitation (AR), often decades earlier than in normal tricuspid valves [14, 15]. Given that strain plays a central role in regulating cellular responses and long-term tissue remodeling, accurate patient-specific quantification of leaflet strain is critical. Specifically, strain can reveal disease progression and identify tissue regions at highest risk for dysfunction or failure. Although not yet part of routine clinical practice, patient-specific strain assessment could ultimately guide surgical decisions toward more durable repairs and reduced need for reintervention.

Despite this clear clinical need for precise and reliable strain assessment, in vivo strain estimation remains a significant unsolved challenge. Clinical imaging modalities, including 4D computed tomography (CT) and 4D echocardiography, lack sufficient temporal and spatial resolution to capture the extremely rapid and large geometric changes that occur during valve opening and closure [16]. Because conventional image-based registration algorithms fundamentally rely on incremental motion between sequential frames, the sparse sampling during fast and large deformation phases leads to substantial tracking errors. Additionally, limited soft tissue contrast and acoustic shadowing further degrade the accuracy of deformation estimates [16]. As a result, existing registration approaches struggle to propagate leaflet geometry across the full cardiac cycle and cannot reliably compute physiologically meaningful strain fields.

Since image-based tracking alone cannot recover full-cycle deformation, finite element (FE) and fluid–structure interaction (FSI) modeling offer a complementary path forward, though these approaches carry their own significant limitations. Prior computational studies have provided important insights into aortic valve leaflet mechanics and hemodynamics [11, 13, 17-21]. Labrosse et al. used 3D+t TEE to construct subject-specific FE models of normal aortic valves and simulate valve function to estimate leaflet stresses [17]. Rego et al. proposed a patient-specific computational pipeline to quantify leaflet deformation in adult trileaflet and bicuspid aortic valves using real-time 3D echocardiography [13]. Their approach relied on manual segmentation of open and closed valve configurations and employed FE-based closure simulations to map the open-state geometry to the imaged closed state, with pressure loading and corrective forces iteratively optimized to achieve shape matching. More recent CT-based approaches have enabled semi-automated reconstruction of patient-specific volumetric aortic valve geometries from angiographic images [19]. In that work, volumetric leaflet models were constructed for a cohort of 25 pediatric patients; however, FSI simulations were demonstrated for only a single representative case and were primarily used to illustrate flow waveforms, without quantitative evaluation of leaflet deformation or patient-specific strain. Despite these advances, most FE and FSI frameworks rely on idealized or parametric geometries [11, 21], simplified boundary conditions [20], and high computational cost, and are often evaluated in small cohorts or single representative cases. Recent efforts have begun to explore tighter integration between biomechanics and image analysis. Wu et al. proposed a framework in which FE-simulated intermediate states were used to augment deep learning-based deformable image registration, demonstrating feasibility on 2D echocardiographic images of three pediatric tricuspid valves [22]. While promising, this initial example was limited to demonstrating shape matching across cardiac phases in two-dimensional images and did not incorporate strain quantification.

Forward FE and FSI simulations can estimate full-cycle valve deformation but cannot fully account for patient-specific variability in geometry, material properties, and boundary conditions. Image-based tracking, meanwhile, cannot capture the rapid motion of valve leaflets between frames. These complementary limitations suggest a natural integration: FE simulation can generate mechanically consistent intermediate deformation states missing from sparse imaging sequences, while image registration can correct deviations arising from modeling assumptions. Building on this idea, we introduce a patient-specific computational framework that couples finite element simulation with deformable image registration. The proposed approach relies only on manual segmentation of the open valve configuration to construct the FE model and uses this model to generate biomechanically informed intermediate configurations, facilitating robust alignment between anatomically distant structures at two time frames, such as open and closed reference AV configurations. Image registration, in turn, corrects deviations arising from modeling assumptions and ensures that leaflet geometry accurately reflects patient-specific imaging data. By combining the strengths of mechanics-based modeling and image-based registration, this framework enables robust tracking of aortic valve deformation and quantitative computation of leaflet strains with bounded uncertainty, addressing the limitations of imaging-only tracking and partially compensating for the modeling assumptions that limit simulation-only approaches. We further evaluate registration accuracy and compute strain distributions across multiple imaging modalities, including 4D CT and 4D TEE, and across a diverse cohort of adult trileaflet, adult bicuspid, and pediatric aortic valves. This framework supports patient-specific biomechanical assessment by providing bounded-uncertainty leaflet strain estimates with the potential to inform tissue-level risk assessment, disease progression, and the generation of complementary training data for learning-based methods.

## Materials and Methods

**Fig. 1:**
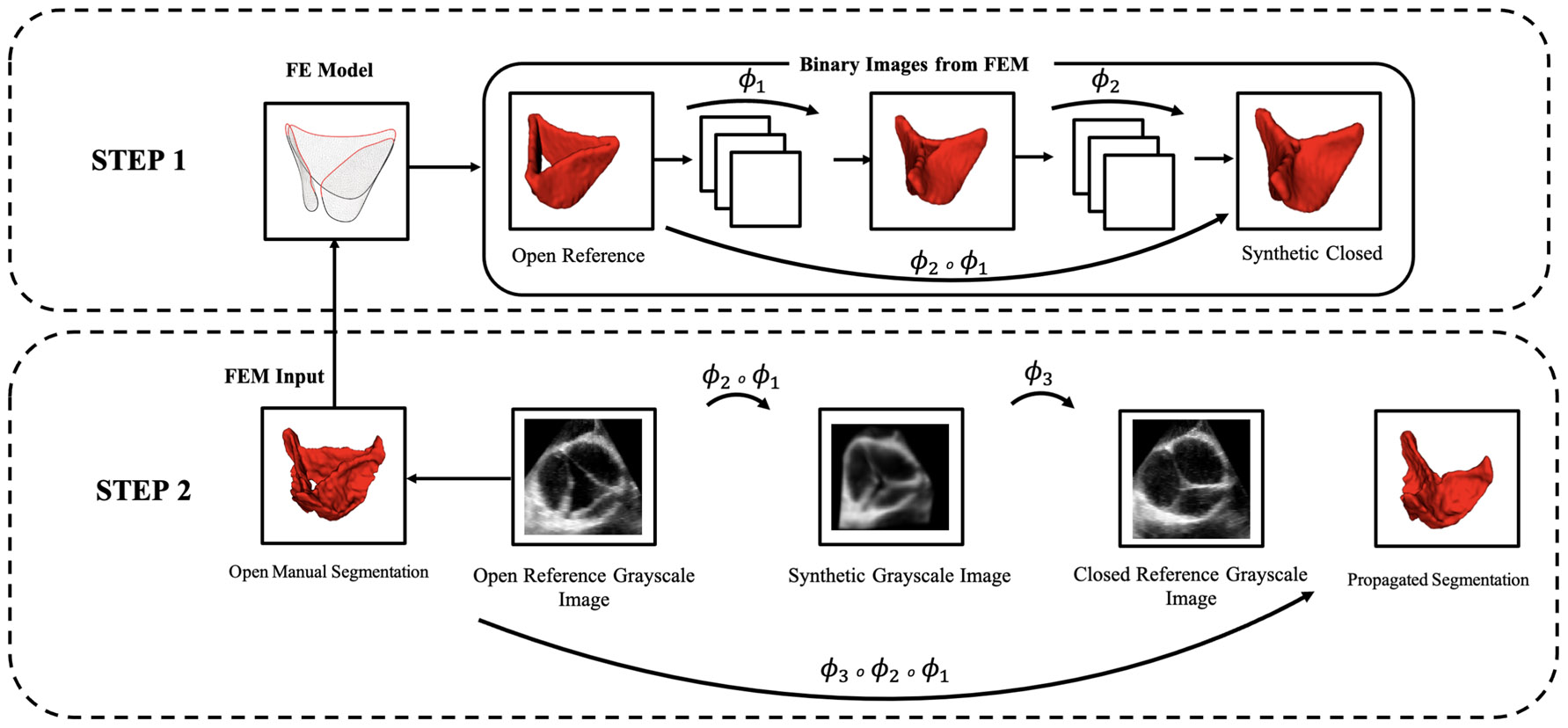
Registration pipeline with binary images from FE simulation

Conventional registration methods cannot accurately align the aortic valve between its two extreme configurations, the open reference configuration at maximum leaflet opening and the closed reference configuration at maximum leaflet coaptation, because the valve’s complex geometry undergoes rapid motion, large non-linear and heterogeneous deformation, and substantial shape changes throughout the cardiac cycle. As a result, conventional feature-tracking approaches have difficulty obtaining anatomically consistent models throughout the cardiac phases. To address this limitation, we proposed a hybrid framework that integrated biomechanical simulation using an FE model with image-based registration, as shown in Fig. 1.

The process begins with the manual segmentation of the aortic valve in the open reference configuration. In the first step, the medial surface of the valve leaflets is extracted from the segmentation of open reference configuration and used to generate a finite element model to simulate valve closure. To characterize the biomechanical behavior of the valve tissue under physiological loading, material properties and diastolic pressure values were obtained from literature and the patient database (when available). Furthermore, physiologically informed boundary conditions obtained from direct registration and were applied to the leaflets to simulate the valve’s deformation from the open reference configuration to the closed reference configuration. The simulation generates a sequence of intermediate geometries that approximate the valve’s deformation between cardiac phases, from the open reference configuration toward the closed reference configuration, which we refer to as the “synthetic closed” state. In the second step, these intermediate geometries serve as prior knowledge within the registration pipeline, guiding the propagation of the open reference segmentation across 4D TEE or 4D CT images. As a result, we obtain an image-driven model of the aortic valve and can evaluate the strain throughout the cardiac phases. In the following, we go through these steps in detail.

### Model Preparation from Clinical Images

#### Segmentation

An accurate geometric representation of the aortic valve is a crucial step in developing a physiologically relevant computational model for biomechanical simulation and deformation analysis. In this study, manual segmentation was used to extract the aortic valve anatomy from 4D TEE or 4D CT images acquired throughout the cardiac cycle. In total, we curated 14 4D TEE datasets and 6 4D CT datasets, 4 of which were from pediatric patients. The collection and analysis of these data were approved by the Institutional Review Boards at the Children’s Hospital of Philadelphia and the University of Pennsylvania.

Two frames were selected for segmentation: (1) maximum leaflet opening (open reference configuration), occurring near peak systole when the leaflets are fully separated; and (2) maximum leaflet coaptation (closed reference configuration), occurring in early diastole shortly after valve closure, when the leaflets are fully in coaptation. The closed reference configuration was chosen because it corresponds to peak transvalvular loading. At valve closure, aortic pressure remains near its early diastolic level ( 80 mmHg), while left ventricular pressure rapidly falls during isovolumic relaxation, creating the largest pressure gradient across the closed leaflets. As diastole continues, this gradient decreases due to declining aortic pressure and rising left ventricular filling pressure [23, 24]. This relationship is also illustrated in the Wiggers diagram [25], where aortic valve closure occurs when the pressure difference between the aorta and left ventricle is maximal in early diastole. Prior studies similarly use the fully coapted state for strain analysis [13, 26]. Both frames are clearly identifiable in the 4D image sequence at the available temporal resolution [27] and represent the extremes of valve deformation, providing suitable reference states for simulation and registration.

Although ML-based segmentation methods have demonstrated good performance in many cardiac imaging applications, their accuracy depends heavily on image characteristics and on training data that reflect similar anatomical features and acquisition settings. To ensure the highest level of accuracy and consistency in the geometric models used for our analysis, we elected to perform manual segmentation for all patients.

Manual segmentation was performed using ITK-SNAP and 3D Slicer, two widely used software platforms for anatomical image labeling [28-30]. The open-frame segmentation served as input for extracting the leaflet surface and developing the FE model, while the closed-frame segmentation served as a reference or “ground truth” for validating propagated segmentations. Because the spatial resolution was limited by the original image acquisition, careful attention was required during segmentation to preserve anatomical details. To preserve key morphological features such as leaflet curvature, commissures, and attachment regions, each frame was manually traced slice by slice across multiple 2D planes, carefully reviewed in orthogonal views, and processed with minimal smoothing to keep small anatomical details accurate. Label maps were then interpolated into 3D volumes, with anatomical landmarks such as the valve annulus and sinus borders guiding the contours to maintain consistency across the two frames. Segmentation was performed under the supervision of a clinical expert to reduce operator bias and ensure accurate representation of the native leaflet anatomy for physiologically realistic simulations. Each segmentation also passed through multiple rounds of verification involving more than one annotator. Additionally, the reproducibility of this 3D TEE segmentation method was evaluated through an inter-observer study on a subset of five patients from the adult 4D TEE cohort (2 TAV and 3 BAV). Two experts independently segmented the aortic valve in both open reference and closed reference frames, and the inter-observer mean distance, averaged across configurations, was 1.32 ± 1.04 mm for the left coronary leaflet, 0.85 ± 0.59 mm for the non-coronary leaflet, and 0.99 ± 0.56 mm for the right coronary leaflet (~1.05 mm averaged over leaflets). The final open-frame label maps were converted into surface meshes for further modeling steps.

#### Medial Surface Extraction

**Fig. 2:**
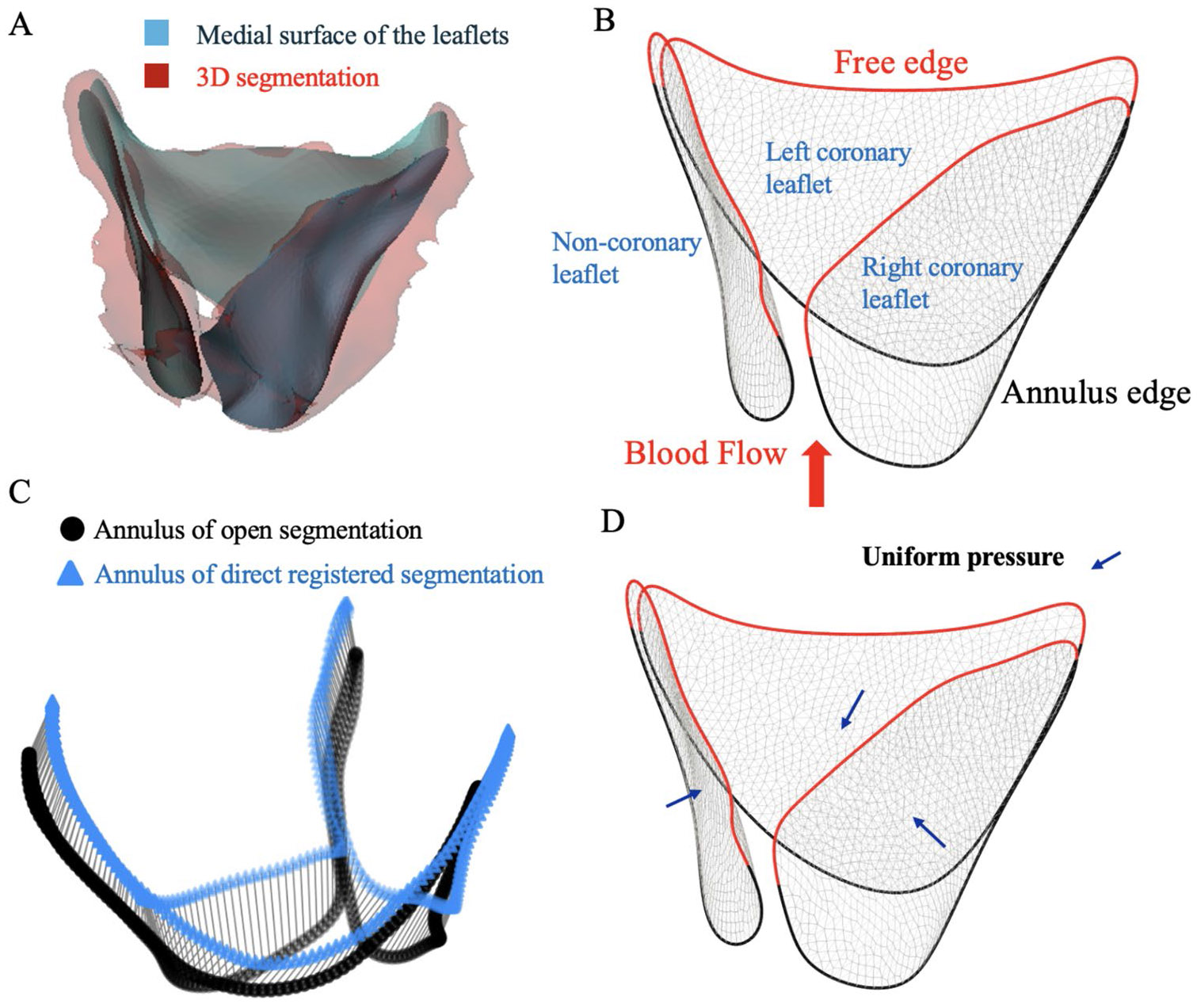
Finite element model development. **A** Extraction of medial surface of the valve leaflets in the open reference configuration **B** Definition of boundary conditions for the annulus and the free edge of the leaflet. **C** Estimation of annulus displacement. **D** Imposed uniform diastolic pressure on the leaflets

After segmenting the valve in the open reference configuration, the medial surface was extracted from the segmentation to serve as the input geometry for FE analysis (Fig. 2A). The medial surface, representing the mid-surface of the valve leaflets, was generated using an interactive skeletonization approach applied to the surface mesh of the binary segmented volume, implemented in 3D Slicer [31]. For completely fused leaflets, the two leaflets were modeled as a single entity to maintain geometric consistency in the medial surface representation.

The medial surface reduces the computational complexity of the FE model while preserving essential anatomical features such as leaflet curvature, commissures, the annular boundary, and the free edges. It also provides a mid-surface reference, from which thickness profiles can be defined across the leaflet to generate shell elements that accurately reflect the leaflet’s 3D geometry and mechanical behavior.

Post-processing of the medial surface included smoothing with preserved boundaries, which were carried out using the FEBio software [32]. The result was a continuous, smooth 3D surface that accurately captured the valve geometry in the open reference configuration. This process ensures that the mesh topology is suitable for finite element simulation. This surface was later used to generate a shell-based finite element model, which serves as the initial configuration in simulating valve closure (Fig. 2B).

### Finite Element Valve Model

#### Constitutive Model

The non-linear, nearly-incompressible mechanical behavior of aortic valve tissue was modeled using a hyperelastic constitutive model. Specifically, we employed the isotropic Lee–Sacks model, a Fung-type exponential strain-energy function previously developed for heart valve simulations [33, 34]. This model combines a neo-Hookean matrix with a Fung-type exponential term to capture the non-linear response of soft biological tissues under large deformations. In addition, it separates the contributions of isochoric deformations and volumetric deformations, allowing the energy associated with shape changes ($\Psi_{\text{isochoric}}$) and volume changes ($U_{vol}$) to be considered independently. The total strain-energy density can be expressed as,

(1) $$\Psi (C,J)=\Psi_{\text{isochoric}}(c_{0},c_{1},c_{2},I_{1})+U_{vol}(J)$$

where the isochoric strain-energy density defined as,

(2) $$\Psi_{\text{isochoric}}=\frac{c_{0}}{2}(I_{1}-3)+\frac{c_{1}}{2}\left(e^{c_{2}{\left(I_{1}-3\right)}^{2}}-1\right).$$

C is the right Cauchy-Green deformation tensor representing the full deformation, $I_{1}=\text{tr}(C)$ is its first invariant, and J=det(F) denotes the local volumetric change. The parameter $c_{0}$ represents the shear modulus of the neo-Hookean matrix, which governs the baseline linear elasticity of the tissue. The parameters $c_{1}$ and $c_{2}$ control the magnitude and rate of exponential stiffening, respectively, and are key to capturing the non-linear behavior of collagenous soft tissues.

For these materials, the entire bulk (volumetric) behavior is determined by the function $U_{vol}(J)$. This function is constructed to have a value of 0 for J = 1 and to be positive for all other values of J>0. In quadratic form, it can be written as $U_{vol}=\frac{k}{2}{\left(J-1\right)}^{2}$, where k is the bulk modulus, controlling the tissue’s resistance to volumetric changes and ensuring near-incompressibility, which is a common property of biological soft tissues.

The material parameters used in this study were k = 5000 kPa, $c_{0}$ = 67 kPa, $c_{1}$ = 13 kPa, and $c_{2}$ = 38 . These values were adopted from prior heart valve studies [35], where the Lee–Sacks model parameters were obtained by fitting equibiaxial experimental data [33, 36]. These parameters are derived from adult bioprosthetic and native leaflet tissue, and the same nominal values were applied across all cases in the cohort (adult trileaflet, adult bicuspid, and pediatric). However, pediatric tissue is generally more compliant and exhibits different collagen fiber architecture, while diseased adult valves (e.g., severely calcified bicuspid or fibrotic valves) may deviate from these baseline properties. Patient-specific parameter tuning was not performed. Instead, the effect of material stiffness on the framework was evaluated through a sensitivity analysis (“”Sensitivity Analysis and Strain Verification” and “Effects of FE Modeling Assumptions on Results” sections).

While the Fung-type model offers realistic mechanical behavior, it is known to introduce convergence challenges under certain loading conditions. Kamensky et al. [37] reported that the exponential term may cause these convergence difficulties, and therefore, they introduced a tangent scale factor which can improve numerical stability. In our simulations, a tangent scale factor of one was applied (TF = 1), but model convergence was monitored carefully to mitigate any divergence issues.

Although recent studies such as [38] have highlighted the importance of anisotropic behavior in valve leaflets due to collagen fiber alignment, we assumed isotropic material behavior for the sake of simplicity. It is acknowledged that this assumption introduces some degree of approximation, particularly in commissural regions where fiber orientation affects local deformation. However, in this study, the FE simulation is not intended to predict exact valve deformation but rather to generate intermediate valve configurations that serve as a structural prior for guiding the registration process. We assume that minor inaccuracies introduced by the isotropic assumption and uniform material properties for all the leaflets are compensated during the image-based registration step.

#### Boundary Conditions and Loading

Physiological boundary conditions (annulus movement and pressure loading on the valve leaflets) were applied to the open reference valve shell model to approximate the nearly closed configuration, referred to as the "synthetic closed" state. In particular, the annulus displacement during the cardiac phases was applied to the annulus to capture the dynamics of the aortic root. This displacement was obtained in a patient-specific manner from initial direct image registration. First, we directly registered the open reference configuration grayscale image to the closed reference configuration image and propagated the open reference configuration segmentation accordingly to obtain the segmentation in the closed reference configuration. Therefore, we can approximate the annulus displacement between the two configurations (Fig. 2C) and impose it in our FE simulation. The free edges of the valve leaflets were left unconstrained.

Furthermore, pressure loading on the leaflets was applied to simulate diastolic closure. The transvalvular pressure gradient for AV, defined as the pressure difference between the left ventricle (LV) and the aorta (AO), ($P=P_{LV}-P_{AQ}$), was reported over a cardiac cycle of 0.76 s [39, 40]. During the opening phase only a small ventricular side pressure (~ 4 to 0 mmHg) acts on the leaflets; once closure begins, aortic side pressure rises sharply to 80–100 mmHg.

Simplified FE models that apply uniform pressure are known to overestimate leaflet stresses compared with fully coupled FSI simulations [20]. For a typical adult diastolic pressure of 80 mmHg, FSI results suggest that only 90–95% of this load is transmitted to the leaflets due to fluid cushioning and coaptation effects [20]. Guided by this range, we applied a uniform nominal aortic side pressure of 75 mmHg across all 16 adult cases (Fig. 2D). Given cohort heterogeneity (bicuspid, calcified, and regurgitant valves), this value is not patient-specific: lower diastolic pressures are expected in moderate-to-severe aortic regurgitation, while higher values may occur in other subgroups [41, 42]. Because the same nominal pressure was applied uniformly across all adult cases, this may introduce geometric discrepancies relative to patient-specific conditions. However, we expect that the subsequent image-based registration step aligns the simulated leaflet closed configuration (synthetic closed) with patient-specific anatomy image, correcting most of these differences. The impact of the uniform pressure assumption on registration accuracy and strain estimates was quantified through a sensitivity analysis (“Sensitivity Analysis and Strain Verification” section). For pediatric cases, a nominal pressure of 45 mmHg was used based on measured pre-CT diastolic values.

#### Simulation and Model Convergence

The finite element model was solved using the dynamic implicit solver of FEBio [32]. The valve leaflets were modeled using linear triangular shell elements with a uniform thickness of 1.2 mm for adult patients and 1 mm for pediatric patients. The thickness value for aortic valve was obtained from [43], where they reported mean thicknesses for aortic valve for different age groups. The simulations were performed multiple times on a single valve model using different mesh densities to assess convergence behavior. Mesh sizes were systematically reduced, and convergence in maximum displacement was achieved with a target edge length of 0.4 mm for the triangular elements, corresponding to a total of 8271 elements. The simulation generated a series of intermediate frames between the open and closed reference configurations, and approximated the "synthetic closed" configuration, which was exported to guide registration. To ensure reproducibility of the simulations, all model parameters are summarized in Table 1.

### Image Registration

Standard image registration methods struggle with the large deformation of the aortic valve between systole and diastole, leading to misaligned propagated segmentations. To address this, we developed a biomechanically informed registration framework to improve robustness in tracking the aortic valve feature across cardiac phases. First, FE simulations generate a sequence of intermediate valve configurations as binary images; second, these intermediate frames are used to guide the registration (see Fig. 1).

In the first step, given the open reference manual segmentation, we develop a medial surface of the valve [31] and simulate valve closure. This simulation brings the aortic valve toward its closed reference configuration, which we refer to as the “synthetic closed” state. From the FE simulation, we extract three frames: the initial, middle, and the final simulation frame, and convert these into binary images. These binary images are then resliced and resampled into the grayscale image space. Then, pairwise registration between the frames is performed to compute the transformations that map one configuration to the next. The composed transformation ($\phi_{2}\circ \phi_{1}$) therefore maps the open reference segmentation to the synthetic closed configuration.

In the second step, this FE-derived transformation ($\phi_{2}\circ \phi_{1}$) is applied to the open reference grayscale image to obtain a synthetic closed image that closely approximates the actual closed reference image. This facilitates the final registration from the synthetic closed to the actual closed reference grayscale image ($\phi_{3}$). The full composed transformation ($\phi_{3}\circ \phi_{2}\circ \phi_{1}$) is then used to propagate the manual open reference segmentation into the closed reference configuration.

The registration was performed between the FE frames using affine and deformable registration with Greedy [44] to obtain a composed transformation mapping the open configuration to the closed configuration. For affine registration, Greedy optimized an affine transformation matrix, initialized by matching the centers of the fixed and moving images (-ia-image-centers), using a sum of squared differences (SSD) metric for segmentation and a normalized cross-correlation (NCC) metric for grayscale images. The NCC metric used a local kernel (radius 2 voxels), and the registration employed a multi-resolution schedule of 100×50× 10 iterations across three resolution levels (coarse, intermediate and full resolution). For deformable registration, Greedy computed a dense displacement field (warp), initialized from the affine result (-it affine. mat), optimized using the same multi-resolution schedule, SSD or NCC depending on the image type, and a stationary velocity field model with Lie bracket correction (-svlb) which applies a more precise update to the velocity field, improving the accuracy of the resulting deformations. The computed warp and affine transformations were then used to resample both grayscale images and segmentation maps into the fixed frame, with label interpolation (-ri LABEL 0.2vox) applied to segmentations to minimize artifacts along boundaries.

This registration process improves the alignment of the propagated segmentation with the patient anatomical images and partially compensates for deviations arising from simulation assumptions. The goal is to enable the construction of a precise, image-driven valve model throughout the cardiac phases and the bounded-uncertainty estimation of leaflet strain.

### Strain Calculation

We compute the total strain on the leaflet for each subject by summing the strains from both steps. For the first step, we compute both the areal strain and the Green–Lagrange strain at the synthetic closed configuration obtained from the FE simulation. The areal strain for each triangular element in the FE mesh is defined as the relative change in its surface area between two configurations,

(3) $$\varepsilon_{A}=\frac{A_{d}-A_{r}}{A_{r}},$$

where $A_{r}$ is the area of the element in the reference configuration (open manual segmentation) and $A_{d}$ is the area of the same element in the deformed configuration (synthetic closed). For the second step, we compute the deformation between the synthetic closed and the closed configuration. The total areal strain is obtained as the sum of the areal strain components from both steps,

(4) $$\varepsilon_{A,\text{total}}=\varepsilon_{A,1}+\varepsilon_{A,2}.$$

The Green-Lagrange strain tensor E is computed from the deformation gradient as,

(5) $$E=\frac{1}{2}(F^{T}F-I),$$

where F is the deformation gradient computed within the FE solver, and I is the identity tensor. However, to compute the Green–Lagrange strain in the second step, we calculate the in-plane Green–Lagrange strain directly from the nodal coordinates of the shell meshes in the two configurations. In the first step, the FE simulation deforms the leaflets from the open reference configuration to the synthetic closed state approximating the closed reference configuration, during which most of the deformation occurs, particularly out-of-plane bending. In the second step, the leaflets are already closed, and only small deformations, mainly in-plane, arise from the registration process, which corrects the leaflet geometry and aligns it with the patient-specific anatomy. Because the deformations in the second step are small, it is reasonable to approximate the strain as in-plane, and this assumption is verified. Finally, the total Green–Lagrange strain is obtained by summing the FE-derived Green–Lagrange strain from the first step with the in-plane strain from the second step,

$$E_{\text{total}}=E_{\text{FE}}+E_{2D}^{\left(3D\right)},$$

where

$$E_{2D}^{\left(3D\right)}=\left[\begin{array}{ccc} E_{xx} & E_{xy} & 0\\ E_{xy} & E_{yy} & 0\\ 0 & 0 & 0 \end{array}\right].$$

The effective (von Mises–type) strain, a scalar measure of deformation, is computed from the total strain tensor $E_{\text{total}}$ as,

$$\varepsilon_{\text{eff}}=\sqrt{E^{\prime }\;:\;E^{\prime }},$$

where

$$E^{\prime }=E_{\text{total}}-\frac{1}{3}\text{tr}(E_{\text{total}})I,$$

is the deviatoric part of the strain tensor. In addition, the Green–Lagrange strain magnitude, which provides an overall measure of local deformation including volumetric changes, is defined as

$$\varepsilon_{\text{GL}}=\sqrt{E_{\text{total}}\;:E_{\text{total}}}.$$

Together, these scalar measures allow quantification of both deviatoric (shape-changing) and total (overall) deformation in the aortic valve tissue.

Finally, strain distributions were visualized using colormaps overlaid on the medial surface mesh, enabling the identification of regions of elevated strain, particularly near the commissures and coaptation lines. In this study, the open reference AV configuration was assumed to be the reference configuration and considered as strain-free, and strains were computed between the two extreme deformation states of the valve, namely the open and closed reference configurations.

### Sensitivity Analysis and Strain Verification

To quantify the influence of finite element modeling assumptions on registration accuracy and the resulting strain estimates, a systematic sensitivity analysis was performed on an adult trileaflet aortic valve case from the TEE dataset (with no aortic regurgitation and no aortic stenosis). Three primary modeling parameters were varied across physiologically plausible ranges:

- *Diastolic pressure* (P): 60, 75 (nominal), 90 mmHg. This range spans effective leaflet loading in adult patients, from moderate aortic regurgitation (where retrograde flow reduces effective diastolic leaflet pressure [20, 41]) to hypertensive loading, covering the hemodynamic variability of the adult cohort.
- *Leaflet thickness* (h): 1.0, 1.2 (nominal), 1.4 mm. These values span the reported range of adult aortic valve leaflet thickness based on the autopsy study by Sahasakul et al. [43], covering the variability across the adult cohort.
- *Exponential stiffness coefficient* ($c_{1}$): 6.5, 13 (nominal), 26 kPa. Among the parameters of the isotropic Lee–Sacks model (“Constitutive model” section), we selected $c_{1}$ because its exponential term $\frac{c_{1}}{2}[\text{exp}(c_{2}{\left(I_{1}-3\right)}^{2})-1]$ dominates the strain-energy response at the moderate-to-large strains that occur during valve closure, while the neo-Hookean matrix term ($c_{0}$) contributes only a linear background. Thus $c_{1}$ is the primary determinant of overall tissue stiffness under physiological loading. This is consistent with the findings of Wu et al. [45], who performed a systematic sensitivity analysis of all Lee–Sacks parameters (using the same isotropic formulation and ±50% perturbation) on image-derived atrioventricular valve models and found that variations in $c_{0}$ had a comparatively smaller influence on the resulting stresses than the exponential parameters. The lower bound (6.5 kPa) represents more compliant effective tissue behavior, such as that observed in pediatric valves, while the upper bound (26 kPa) represents stiffer effective tissue behavior, such as that observed in aged or fibrotic adult leaflets. The pressure and thickness ranges apply to the adult cohort; pediatric cases use separate nominal values (45 mmHg and 1.0 mm, Table 1), while the material stiffness range (− 50 to +100% of nominal) intentionally spans both populations.

A one-factor-at-a-time (OFAT) design augmented with two corner runs was employed, requiring a total of 9 FE simulations. The two corner runs bound the extreme parameter combinations: a compliant corner ($c_{1}$ = 6.5 kPa, h = 1.0 mm, P = 60 mmHg) and a stiff corner ($c_{1}$ = 26 kPa, h = 1.4 mm, P = 90 mmHg). This design identifies the individual effect of each parameter, while the corner runs test the extreme combinations. For the compliant corner run, the FEBio simulation did not reach the prescribed target pressure and terminated at 43 mmHg (71% of the 60 mmHg target) due to convergence difficulties inherent to the combination of a low material stiffening and thin leaflet.

For each of the 9 configurations, the FEM-generated intermediate configurations were used to guide the complete registration pipeline. Registration accuracy was quantified using the mean distance between the propagated closed segmentation and the ground-truth manual segmentation. For each run, the total strain was decomposed into two sequential steps as described in “Strain Calculation” section: Step 1 (FE simulation: open reference → synthetic closed), in which the Green–Lagrange strain tensor $E_{\text{FE}}$ was computed by the FEBio solver from the full-3D deformation gradient and the areal strain $\varepsilon_{A,1}$ was computed from the change in element area; and Step 2 (registration correction: synthetic closed → closed reference), in which the in-plane Green–Lagrange strain $E_{2D}^{\left(3D\right)}$ was computed from the nodal coordinates of the shell mesh and the areal strain $\varepsilon_{A,2}$ was computed from the change in triangle area. To verify the geometric strain computation, both the Green–Lagrange magnitude and the areal strain were additionally computed for Step 1 independently from the mesh coordinates and compared against the FEBio-computed reference values for all 9 configurations.

## Results

We applied the methodology to 20 patients (16 adult and 4 pediatric), including 14 4D TEE and 6 4D CT datasets. These images were acquired intraoperatively or immediately prior to the procedure. Patient characteristics, including valve morphology and severity of aortic regurgitation (AR) and stenosis (AS), are summarized in Table 2. Overall, the combined cohort represented a broad spectrum of valve morphologies and disease severities, encompassing patients with normal to severely regurgitant valves and none to moderate stenosis.

**Fig. 3:**
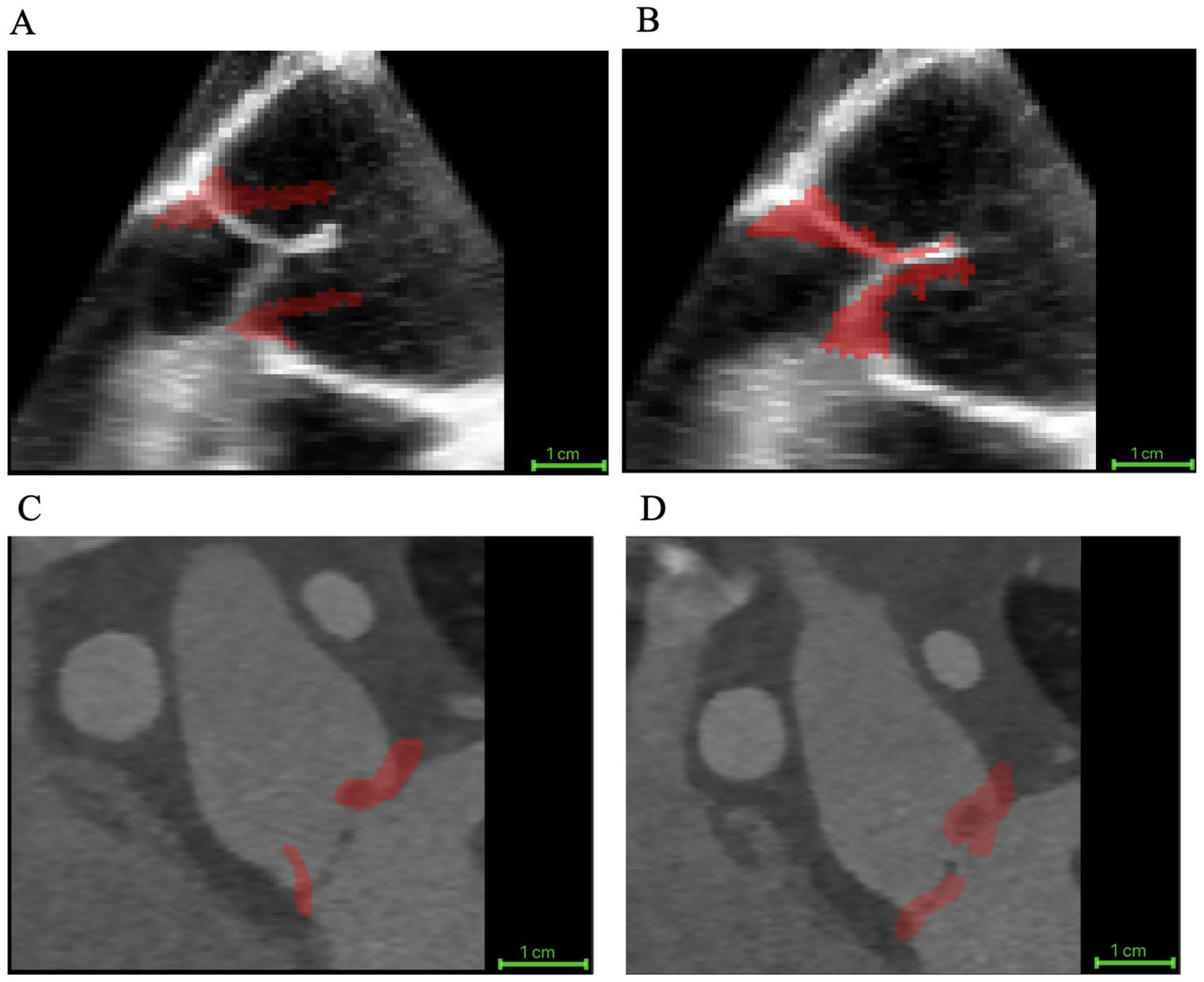
Example of image-based registration results for one TEE case and one CT case. The reconstructed aortic valve leaflets in the closed reference configuration are shown in red. **A** TEE with direct registration, **B** TEE with FEM-augmented registration, **C** CT with direct registration, and **D** CT with FEM-augmented registration

The proposed biomechanically informed registration improved segmentation-tracking accuracy by an average of 40%, reflecting the reduction in the mean distance between the tracked closed-state segmentation and the manual ground truth across all 20 cases when compared with direct registration. Specifically, this mean distance decreased from 3.70 ± 2.30 mm with direct registration (no FEM) to 2.23 ± 1.27 mm using our approach. Table 3 summarizes registration accuracy across valve morphologies and imaging modalities, comparing the mean distance between the manual ground truth and the propagated segmentation obtained with direct registration and proposed FEM-augmented registration. Figure 3 illustrates an example of the reconstructed patient-specific aortic valve closure (shown in red) overlaid on closed reference grayscale images. Panels A and B show a TEE case, and panels C and D show a CT case. For each modality, the left panel displays the result from direct registration, and the right panel shows the result from the FEM-augmented registration method. Furthermore, to assess the biomechanical behavior of different valve morphologies, we evaluated leaflet strain in adult trileaflet, adult bicuspid, and pediatric cases and compared the resulting strain ranges. The resulting strain maps exhibited physiologically consistent patterns.

### Effects of FE Modeling Assumptions on Results

To establish confidence in the proposed framework before presenting the detailed case-specific findings, we characterized the robustness of the FEM-augmented registration and the resulting strain estimates to the FE modeling assumptions. Table 4 summarizes the sensitivity analysis results, showing the registration accuracy and total strain for each of the 9 parameter configurations along with the percentage variation from the baseline. The mean distance between the propagated and ground-truth closed segmentations was 0.904 ± 0.012 mm across all 9 runs, with a maximum deviation of 2.0% from the baseline (0.905 mm), demonstrating that variation in FEM parameters within physiologically plausible ranges has negligible effect on registration accuracy. The total Green–Lagrange strain magnitude ranged from 0.159 to 0.194 (mean 0.174 ± 0.015), with a maximum deviation of 10.6% from the baseline (observed for the $c_{1}$ soft run). The total areal strain ranged from 0.079 to 0.132 (mean 0.103 ± 0.019), with a maximum deviation of 26.1% from the baseline (observed for the stiff corner run), reflecting the larger sensitivity of areal strain to the assumed parameters through the Step 1 FE simulation. While maximum deviations reflect the upper bound, the overall variability is captured by the coefficient of variation (CV = SD/mean × 100) was 1.3% for the registration mean distance, 8.4% for the total Green–Lagrange strain, and 18.4% for the total areal strain.

The variation bounds for each single parameter are as follows. For pressure (±20% perturbation): the mean distance deviated by at most 2.0%, the total Green–Lagrange strain by at most 9.1%, and the total areal strain by at most 13.2% from the baseline. For thickness (±17% perturbation): the mean distance deviated by at most 1.4%, the total Green–Lagrange strain by at most 10.3%, and the total areal strain by at most 24.6%. For material stiffness (− 50 to +100% of nominal perturbation): the mean distance deviated by at most 1.2%, the total Green–Lagrange strain by at most 10.6%, and the total areal strain by at most 14.1%. The two corner runs, which combine extreme values of all three parameters simultaneously, produced mean distances within 1.6% of the baseline; the stiff corner also produced the overall maximum deviation in the total areal strain (26.1%). For the compliant corner run (Run 8), the FEBio simulation did not converge beyond 43 mmHg (71% of the 60 mmHg target); its values are reported in the tables for completeness.

Table 5 presents the strain decomposition into Step 1 and Step 2 components together with the strain computation verification. In Step 1, the Green–Lagrange strain magnitude from the FEBio solver was compared against the in-plane formulation computed from the mesh coordinates. The in-plane formulation systematically underestimated the FEBio reference by a mean of −7.3 ± 1.7% across all 9 runs, with a maximum error of 9.8%. Importantly, for Step 1 the pipeline uses the FEBio-computed strain directly; the in-plane geometric calculation is used only for Step 2, where the valve is already in a near-closed state (synthetic closed) and the deformations during registration are small and predominantly in-plane. The areal strain computed from the triangle areas of the mesh matched the FEBio-computed areal strain exactly (0.0% difference across all 9 runs), confirming that the geometric computation of areal strain from mesh coordinates is exact.

The Step 2 strain was nearly constant across all 9 runs: the Green–Lagrange magnitude ranged from 0.034 to 0.037 (0.036 ± 0.001), and the areal strain ranged from 0.030 to 0.034 (0.033 ± 0.001). Excluding the Corner soft run, the maximum deviation of the Step 2 strain from the baseline was only 5.4% (Green–Lagrange) and 5.9% (areal). To explain this limited variability, the rigid body displacement during registration Step 2 was examined and found to range from 3.7 to 4.7 mm across all runs, whereas the local deformation remained small and nearly constant. This indicates that the registration correction between the synthetic closed and closed reference is predominantly a rigid realignment rather than a local deformation, which explains the observed constancy of the Step 2 strain. It is important to note that during registration, Greedy first applies an affine transformation to align the images and then applies deformable registration.

The total strain variation across the parameter space arises from Step 1, reflecting the sensitivity of the FE-simulated deformation to the assumed material properties, thickness, and pressure. Step 1 strain deviated from baseline by up to 10.8% for Green–Lagrange and 35.6% for areal strain. Crucially, for areal strain, the maximum deviation of the total strain (26.1%) is markedly lower than that of Step 1 alone (35.6%), demonstrating that the nearly constant Step 2 contribution partially compensates for the Step 1 variability and stabilizes the overall areal strain estimate. For Green–Lagrange strain, the total variability (10.6%) is essentially identical to that of Step 1 (10.8%), showing that the registration correction does not introduce any additional variability despite parameter perturbations.

### 4D Echocardiography Images

#### Trileaflet Aortic Valves

##### Patient characteristics

**Fig. 4:**
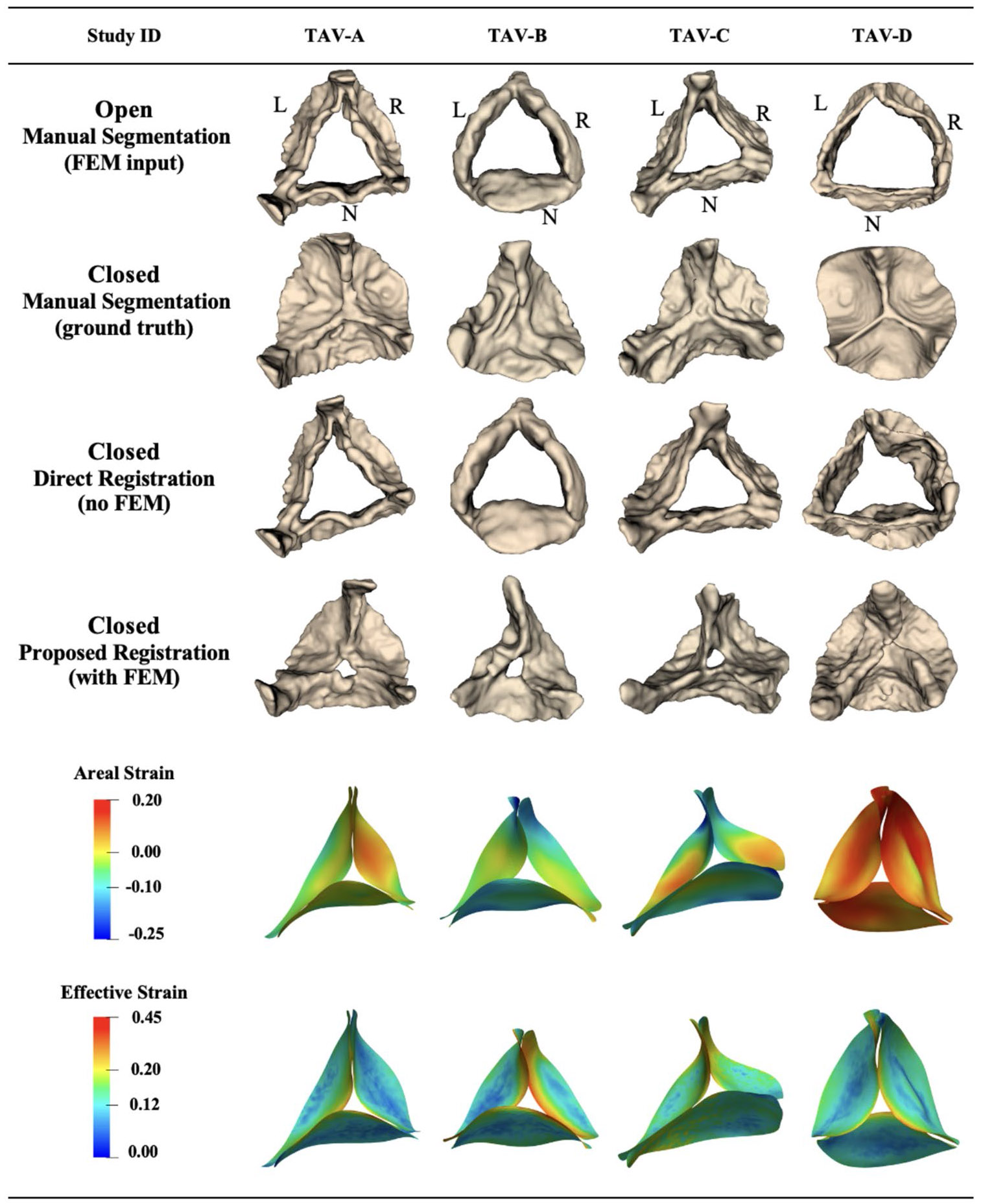
Registration and strain results for trileaflet adult aortic valves from 4D TEE images. Examples with lower (TAV-A/B/C) and higher (TAV-D) mean distance values after valve registration from open to closed reference configuration, computed between registered and ground-truth manual segmentations. L: left coronary leaflet; R: right coronary leaflet; N: non-coronary leaflet

Among 14 adult 4D TEE images, 11 were trileaflet and 3 were bicuspid aortic valves. The mean distance between the propagated segmentation for the closed state and the manual ground-truth segmentation for these 11 patients was 1.68 ± 0.52 mm using the proposed biomechanically informed registration approach and 2.52 ± 0.56 mm with direct registration. Figure 4 shows the propagated segmentation results and strain for four of these cases. Patients TAV-A, TAV-B, and TAV-C were selected to represent some of the best registration results, whereas patient TAV-D was selected to represent one of the worst. These four adult trileaflet valves displayed a spectrum of valve function: normal (TAV-D), mild (TAV-C), mild-to-moderate (TAV-A), and moderate (TAV-B) aortic regurgitation, with no significant stenosis. Each case originated from a distinct clinical context: aortic aneurysm (TAV-A and TAV-C), aortic valve disorder (TAV-B), and coronary artery disease (TAV-D).

##### Registration accuracy

The mean distance deviation from the ground truth for patients TAV-A, TAV-B, TAV-C, and TAV-D was 1.23, 1.27, 2.43, and 2.73 mm, respectively, using the proposed approach. For the same cases, the corresponding values using direct registration were 1.96, 2.08, 2.58, and 3.45 mm. Comparing the two methods, it was clear that direct registration (without FEM) could not fully reproduce leaflet closure. Among the 11 patients, one of the worst propagation results were observed for case TAV-D. Although the proposed method improved the mean distance by 21% for this case, the large and complex deformation combined with limited image quality restricted further improvement in the final registration step. The large deformation in case TAV-D is evident from the comparison of open and closed reference configuration segmentations, as well as the effective and areal strain values (12.2% and 6.9%, respectively), which are also visualized in Fig. 4.

##### Strain

The mean effective Green-Lagrange strain across the leaflets for the representative adult trileaflet cases ranged from 11.7% (TAV-A) to 14.4% (TAV-B), with maximum local strain reaching 36.4% in TAV-D. Maximum effective Green-Lagrange strain primarily occurred near coaptation lines and commissures, consistent with previous studies [11, 13, 20, 46]. The mean areal strain ranged from −4.9% (TAV-C) to 6.9% (TAV-D), with maximum local areal strain reaching 26.5% in TAV-D. Notably, TAV-D, despite a relatively large overall deformation, exhibited a uniform areal strain distribution consistent with normal valve function. In contrast, TAV-B, associated with mild-to-moderate regurgitation, had a lower mean areal strain (3.1%) but a high maximum areal strain (22.1%), reflecting that most of the leaflet surface underwent modest deformation while specific regions experienced localized overextension. Table 6 summarizes the registration accuracy and strain values for these representative cases, providing a quantitative reference for the observations described above.

#### Bicuspid Aortic Valves

##### Patient characteristics

**Fig. 5:**
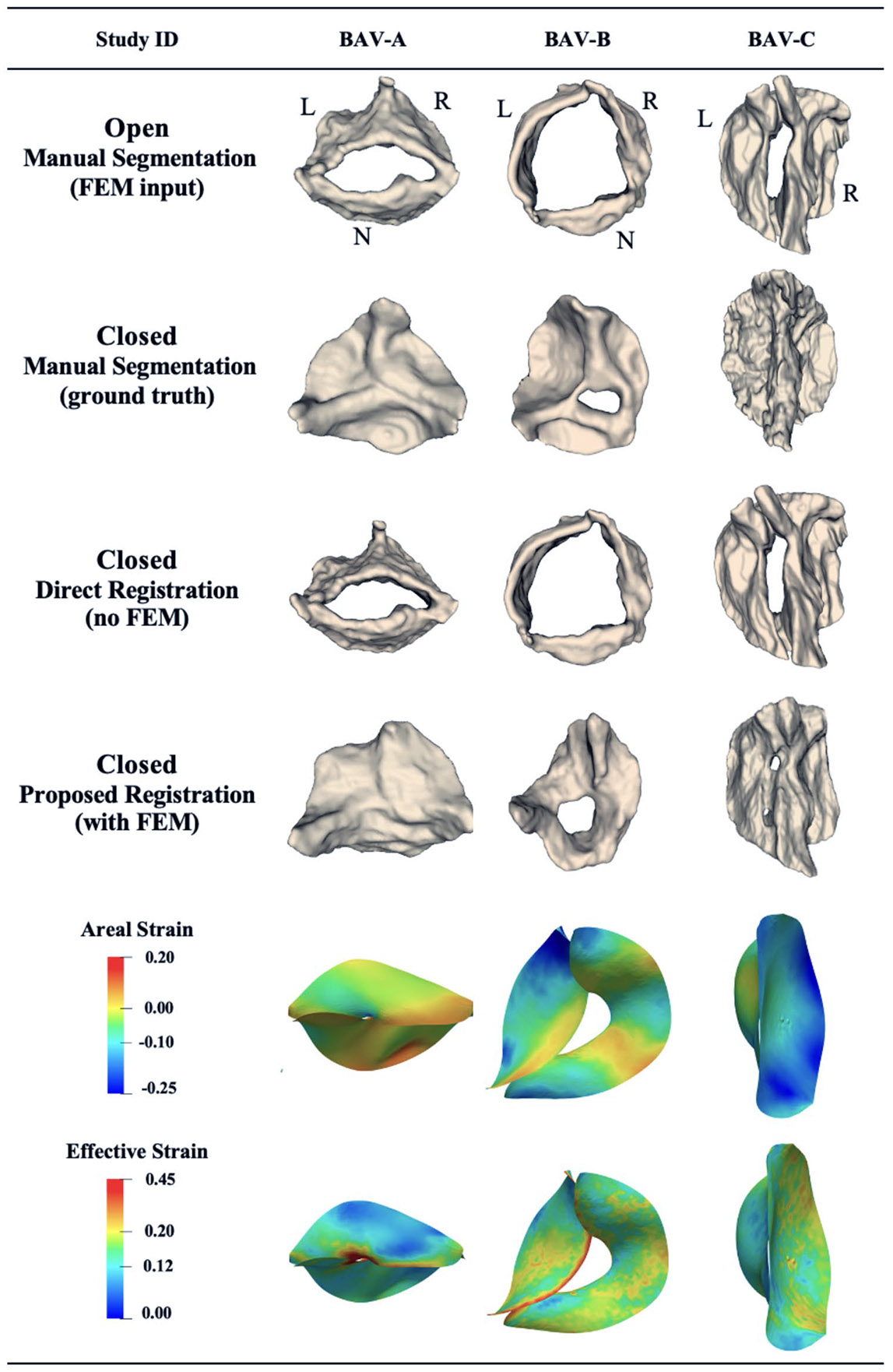
Registration and strain results on bicuspid adult aortic valves 4D TEE images, showing valve registration from open to closed reference configuration. L: left coronary leaflet; R: right coronary leaflet; N: non-coronary leaflet

Among 14 adult 4D TEE images, 3 were bicuspid aortic valves. The mean distance between the propagated segmentation for the closed state and the manual ground-truth segmentation for these 3 patients was 2.04 ± 0.59 mm using the proposed biomechanically informed registration approach and 3.69 ± 0.34 mm with direct registration. Figure 5 shows the propagated segmentation results and strain for these cases. These three adult bicuspid valves exhibited a range of valve function: no aortic regurgitation (BAV-C), mild regurgitation (BAV-A), and severe aortic regurgitation (BAV-B). Aortic stenosis severity varied: absent in BAV-B, mild in BAV-A, and moderate-to-severe in BAV-C. Calcification was present in BAV-C (severe). Each case arose from a distinct clinical context: ascending aortic aneurysm (BAV-A), aortic regurgitation (BAV-B), and combined aortic aneurysm and aortic valve disease (BAV-C).

##### Registration accuracy

The mean distance deviation from the ground truth for patients BAV-A, BAV-B, and BAV-C was 1.62, 1.78, and 2.71 mm, respectively, using the proposed approach, and these metrics for the same cases were 3.38, 3.63, and 4.06 mm using direct registration. Patient BAV-B had severe regurgitation, which can be observed in the propagated segmentation using proposed method in Fig. 5.

##### Strain

Furthermore, the mean effective Green-Lagrange strain across the leaflets for the bicuspid cases ranged from 13.0% (BAV-C) to 14.1% (BAV-B), with maximum local strain reaching 41.9% in BAV-A, reflecting regions of high leaflet deformation during closure (Table 6). The mean areal strain varied more widely, from −14.0% (BAV-C) to −3.2% (BAV-A), with BAV-B showing an intermediate mean areal strain of −10.7% and a high maximum of 17.6%, indicating that most of the leaflet surface underwent moderate compression, while specific regions experienced local overextension. These patterns correlate with valve pathology: BAV-A, with a larger coaptation area, exhibited higher maximum effective strain; BAV-B, with severe regurgitation, showed pronounced local overextension; and BAV-C, with severe calcification, displayed lower maximum strain and uniform compression, consistent with restricted leaflet mobility (Fig. 5).

#### 4D Computed Tomography Images

##### Patient characteristics

**Fig. 6:**
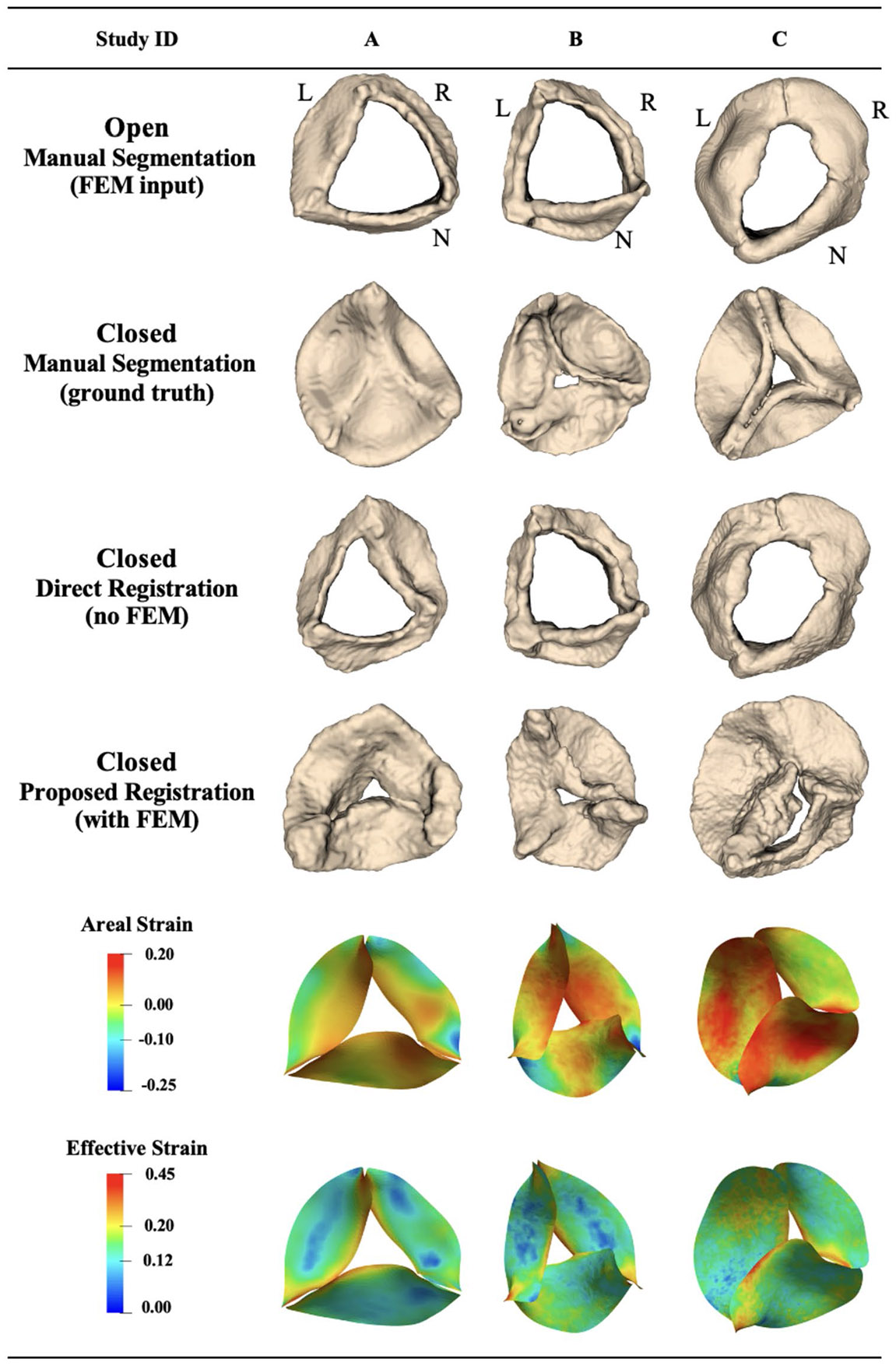
Registration and strain results for pediatric aortic valves from 4D CT images, showing valve registration from open to closed reference configuration. L: left coronary leaflet; R: right coronary leaflet; N: non-coronary leaflet

The 6 patients with 4D CT images included 4 pediatric patients and 2 adults. The mean distance between the propagated segmentation for the closed state and the manual ground-truth segmentation for these 6 patients was 3.49 ± 1.68 mm using the proposed biomechanically informed registration approach and 6.44 ± 2.32 mm with direct registration. Figure 6 shows the propagated segmentation results and strain for 3 pediatric cases. Patients A and B were selected to represent some of the best registration results, whereas patient C was selected to represent one of the worst. These three pediatric aortic valves exhibited a range of function: trileaflet valves (A and B) and a unicuspid valve with partial R/N and R/L leaflet fusions (C). Aortic regurgitation severity ranged from severe (A) to moderate-to-severe (B) and moderate (C). Aortic stenosis was absent in A and B cases and mild in C. Each case arose from a distinct clinical context: severe neo-aortic insufficiency (A), moderate-to-severe neo-aortic regurgitation with moderate left ventricular dilation (B), and combined aortic valve insufficiency and stenosis (C).

##### Registration accuracy

The mean distance deviation from the ground truth for patients A, B, and C using the proposed approach was 1.91, 2.30, and 5.12 mm, respectively. Using direct registration, the corresponding values were 2.97, 4.50, and 9.62 mm. The worst propagation performance was observed for case C (unicuspid). In this case, the mean distance remained high for both methods, indicating inferior image quality. Although the proposed approach improved the mean distance by 47%, the large and complex leaflet deformations limited the accuracy of the FE simulation. Consequently, due to the limited image quality the final registration step also was unable to fully capture the valve motion.

##### Strain

The mean effective Green–Lagrange strain across the pediatric valve leaflets ranged from 15.5% (A) to 15.8% (C), with maximum local strain reaching 45.0% in the unicuspid case C, reflecting extensive leaflet deformation during closure (Table 6). The mean areal strain varied from −1.8% (A) to 2.4% (C), with case C showing the most heterogeneous deformation, including extreme local compression (− 31.1%) and high local expansion (23.3%). In contrast, cases A and B (trileaflet) exhibited moderate areal strain values, with large local minima (−24 to −25.5%) and moderate maxima (15–17%), consistent more uniform leaflet motion. These patterns illustrate that pediatric valves, particularly case C (unicuspid), undergo larger and more nonuniform deformations than adult valves.

### Strain Evaluation on the Leaflets

**Fig. 7:**
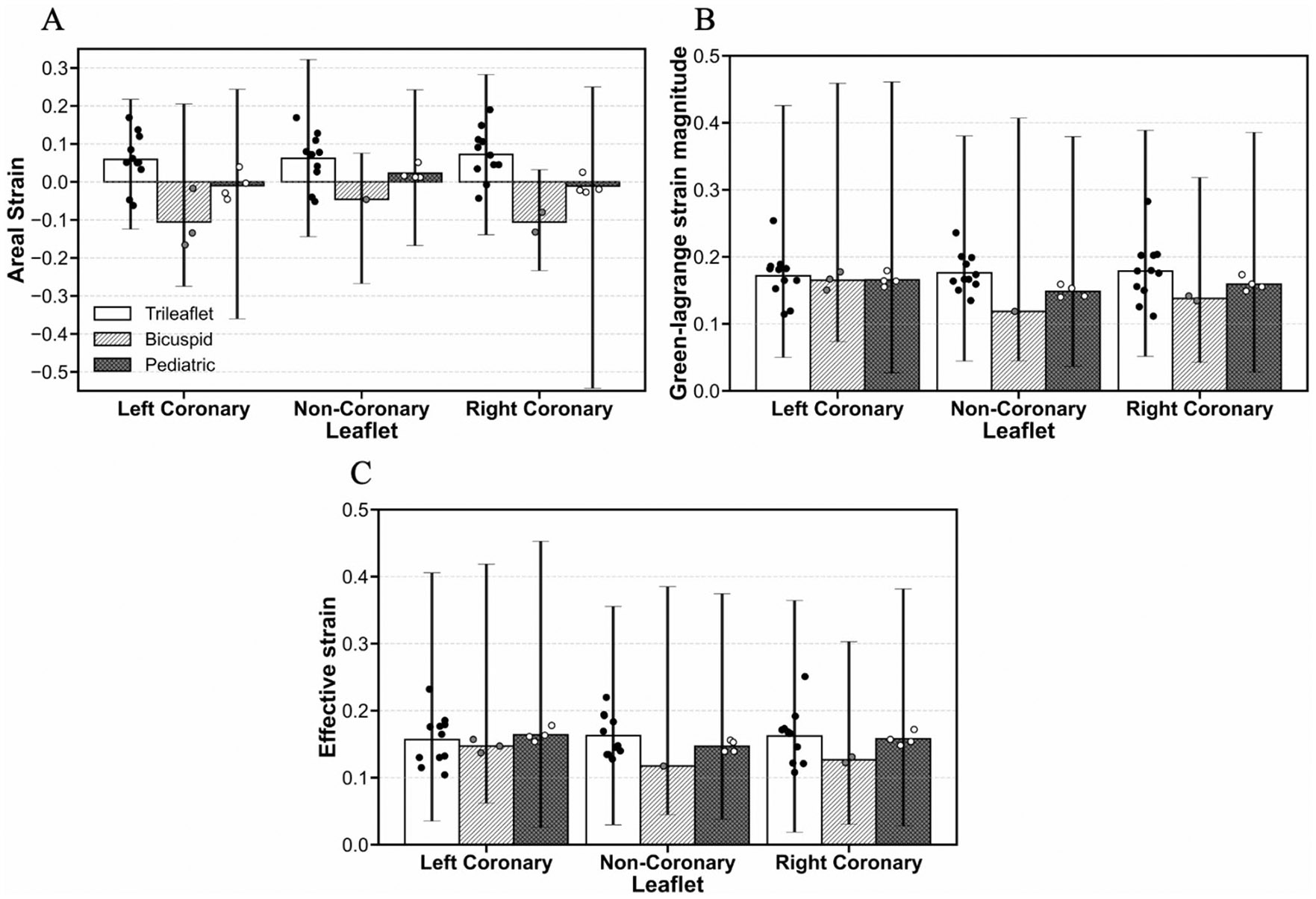
Strain analysis across the aortic valve leaflets (left coronary, non-coronary, and right coronary) in trileaflet, bicuspid, and pediatric valve groups. Bars show the mean strain for each leaflet within each valve group. Vertical ranges indicate the observed minimum–maximum strain across samples for each leaflet within each group. Individual points over each bar represent the mean strain measured on each valve leaflet. (A) Areal strain; (B) Green-Lagrange magnitude; (C) Effective strain

Furthermore, we analyzed areal strain, the magnitude of the Green–Lagrange strain, and effective strain for each aortic valve leaflet (left coronary, non-coronary, and right coronary) in trileaflet adult, bicuspid adult, and pediatric valve groups, as shown in Fig. 7. To facilitate comparison across valve groups and strain metrics, the bars in Fig. 7 represent the mean strain for each leaflet within each valve group, while the vertical lines indicate the observed minimum and maximum strain for each leaflet within each group. Individual points over each bar represent the mean strain measured on each valve leaflet.

The results for areal strain across aortic valve leaflets (left coronary, non-coronary, and right coronary) in trileaflet adult, bicuspid adult, and pediatric valve groups are shown in Fig. 7A. In trileaflet adult valves, mean areal strain was 5.9%, 6.2%, and 7.2% for the left coronary, non-coronary, and right coronary leaflets, respectively. The maximum strain observed in this group was 32.2% and the minimum strain was −14.4%, both on the non-coronary leaflet. In contrast, bicuspid adult valves exhibited lower, predominantly negative areal strains: −10.6% (left coronary), −4.6% (non-coronary), and −10.6% (right coronary). The observed strain range was wide, from −27.5 to 20.5% on the left coronary leaflet, reflecting pronounced heterogeneity. Pediatric valves displayed generally lower areal strains compared with adult groups, with mean values near zero: −1.0% (left coronary), 2.3% (non-coronary), and −1.1% (right coronary). Despite these low mean values, observed strain ranges were wide, particularly for the right coronary leaflet (− 54.3 to 25.0%).

The results for Green-Lagrange magnitude strain, which captures the overall local deformation including both deviatoric and volumetric components, across the same leaflets and valve groups are shown in Fig. 7B. In trileaflet adult valves, mean strains were 17.3%, 17.7%, and 17.6% for the left coronary, non-coronary, and right coronary leaflets, respectively. The maximum observed strain was 42.6% on the left coronary leaflet, and the minimum observed strain was 4.5% on the non-coronary leaflet. Bicuspid adult valves exhibited slightly lower mean strains: 16.5% (left coronary), 11.9% (non-coronary), and 13.8% (right coronary), with a maximum strain of 45.9% on the left coronary leaflet and a minimum of 4.3% on the right coronary. Pediatric valves showed mean strains comparable to adult groups: 16.6% (left coronary), 14.8% (non-coronary), and 15.9% (right coronary). Similar to areal strain, Green-Lagrange strain magnitude exhibited wide ranges, particularly for the left coronary leaflet (2.7 to 46.1%).

Finally, we present the results for effective (von Mises-type) strain, which quantifies primarily deviatoric (shape-changing) deformation, across the same leaflets and valve groups (Fig. 7C). Effective strain values followed patterns similar to those observed for Green–Lagrange magnitude strain but were consistently lower, reflecting the exclusion of volumetric contributions to the total deformation. Importantly, the difference between trileaflet adult and pediatric mean strain was reduced when assessed using effective strain. Effective strain values were similar between the two groups, with trileaflet adult leaflet averages ranging from 15.9 to 16.5% and pediatric valve averages ranging from 14.7 to 16.4%, resulting in minimal group differences. Although the pediatric cohort is small and no definitive conclusions can be drawn, this comparison suggests that age and size-related differences in leaflet deformation are more associated with volumetric deformation than with deviatoric deformation.

## Discussion

Reliable and precise estimation of patient-specific aortic valve leaflet strain is crucial to understand the link between valve biomechanics, disease progression, and tissue remodeling, since abnormal strain patterns accelerate extracellular matrix dysregulation and calcific aortic valve disease, particularly in bicuspid and pediatric valves with congenital defects [4, 6, 11-13]. Despite this clear clinical and mechanobiological importance, in vivo strain estimation remains a significant challenge. Advanced imaging and biomechanical modeling have independently improved our understanding of valve function, but neither approach alone can capture physiologically reliable and precise leaflet deformation. This limitation arises in part because the aortic valve leaflet is a thin, discontinuous structure with free edges that undergoes large, rapid, and heterogeneous deformation. Image-based tracking methods are limited by sparse temporal sampling, low soft tissue contrast, and rapid leaflet motion, which can lead to substantial registration errors [16]. FE and FSI models are constrained by idealized geometries, assumed boundary conditions and material properties, high computational cost, and limited patient-specific validation [13, 17, 20, 21].

To address this, we developed a framework that couples deformable image-based registration with finite element simulation to approximate patient-specific aortic valve closure and quantify leaflet strain with bounded uncertainty. Unlike existing shape matching or FE-based closure frameworks that rely on segmentation of both open and closed reference configurations and iteratively optimize pressure loading and corrective forces to achieve geometric correspondence, the proposed approach requires segmentation of only the open reference configuration. The FE model is used to generate biomechanically informed intermediate valve configurations that facilitate robust alignment between anatomically distant open and closed reference image frames through deformable image registration, thereby eliminating the need for direct shape matching to the closed segmentation. This integrated framework not only improves registration accuracy but also partially compensates for uncertainties in pressure, thickness, and material properties through image-based registration correction, as demonstrated by the sensitivity analysis (“Effects of FE Modeling Assumptions on Results” section), while reducing reliance on fully detailed valve models that require many assumed parameters and incur high computational cost. Therefore, the proposed framework provides a practical pathway toward patient-specific biomechanical characterization of aortic valve function and can generate the complementary data required by learning-based methods.

### Impact of FE Modeling Assumptions on Registration Accuracy and Strain

A central concern for any simulation-informed framework is the sensitivity of its outputs to modeling assumptions. We therefore open this discussion by quantifying how FE modeling choices propagate into the registration accuracy and the resulting strain estimates (“Effects of FE Modeling Assumptions on Results” section). The coefficient of variation was 1.3% for the registration mean distance, 8.4% for the total Green–Lagrange strain, and 18.4% for the total areal strain estimated under physiologically plausible uncertainties in material properties, thickness, and pressure for an adult patient.

The near-constant registration mean distance indicates that the image-based registration step effectively absorbs geometric differences between synthetic closed configurations produced by different FEM parameters and consistently aligns them to the patient-specific imaging data. The analysis of strain and displacement in Step 2 further suggests that this correction is predominantly rigid body realignment rather than local deformation, explaining the near-constant Step 2 strain across all configurations. Note that during registration, Greedy first applies an affine transformation to align the images and then applies deformable registration, which seems prioritize rigid body motion over local deformation.

In contrast, the total strain exhibited measurable variation, with areal strain showing greater sensitivity than Green–Lagrange strain, particularly to leaflet thickness (“Effects of FE Modeling Assumptions on Results” section). This behavior is consistent with thin-membrane mechanics: leaflet stress scales inversely with thickness according to the Law of Laplace σ∼PR∕h [47], and areal strain multiplicatively couples both principal stretches ($\varepsilon_{A}\approx \lambda_{1}\lambda_{2}-1$), so modest changes in thickness produce amplified changes in area deformation. Prior FE studies report similar trends, including substantial stress reductions with increased thickness and large increases with decreased thickness [48, 49], supporting the magnitude of variation observed here. The use of a uniform leaflet thickness follows common practice in aortic valve FE and FSI studies [20, 33, 48-50]. A key motivation is the difficulty of obtaining reliable patient-specific thickness from clinical imaging: leaflet thickness (0.67–1.42 mm across age groups [43]) is on the order of the spatial resolution of echocardiography. As a result, imaging artifacts and resolution limitations (including anisotropic voxel resolution, partial-volume effects and motion artifacts) can cause thin structures to appear artificially thickened, leading to overestimation of leaflet thickness [51, 52]. Adopting a uniform thickness based on anatomical ranges therefore avoids propagating imaging errors into the simulation, while the sensitivity analysis quantifies the associated uncertainty in strain estimates.

These maximum variation bounds should be interpreted as the expected range of strain estimates under physiologically plausible uncertainties in material properties, thickness, and pressure, rather than as a measure of methodological error. As reported in (“Effects of FE Modeling Assumptions on Results” section) the registration correction (Step 2) partially compensates for Step 1 variability in the total areal strain but leaves the total Green–Lagrange strain variability essentially unchanged, confirming that registration does not amplify modeling-induced variation. This difference arises because Step 2 contributes a larger fraction of the total areal strain (~32%) than of the Green–Lagrange strain (~17%), resulting in a stronger stabilizing effect on areal strain.

Finally, the strain verification (Table 5) shows that the in-plane Green-Lagrange approximation systematically underestimates the full FEBio reference strain as it does not account for the out-of-plane components of deformation. However, this does not affect the final total strain results, as the full-3D FEBio strain is used for Step 1 and the in-plane approximation is applied only in Step 2, where deformations are small and predominantly in-plane.

### Image Registration Improvement

Across 20 patients, we observed a 40% improvement in registration accuracy using the proposed FEM-augmented tracking method compared to direct registration. This improvement reflects the reduction in the mean distance between the tracked closed-state segmentation and the manual ground truth across all 20 cases, calculated as the relative decrease compared to direct registration. When analyzed separately, the improvement was 33% for the 14 4D TEE images and 46% for the 6 4D CT images, calculated in the same way for each imaging modality (Table 3). The registration improvement reflects the benefit of incorporating intermediate frames from FE simulation, which were used as binary images to guide registration, whereas direct registration relies solely on image information.

Direct deformable registration was selected as the benchmark to isolate the contribution of the biomechanically generated intermediate frames. Recent learning-based registration methods (e.g., VoxelMorph [53], probabilistic diffeomorphic registration [54], and deep affine–deformable frameworks [55]) perform well on structures with continuous geometry and moderate inter-frame displacement, but the aortic valve is a thin, discontinuous structure with free edges undergoing large, heterogeneous deformation that violates the smooth-deformation assumptions of these frameworks; training also requires large valve cohorts that are not currently available for our patient-specific aortic valve leaflet analysis. The proposed framework is therefore complementary rather than competing with learning-based registration methods: the framework provided reference and intermediate configurations (open → intermediates → closed) can serve as training pairs for future learning-based registration.

The reported mean distance should be interpreted with respect to what the metric measures: a surface-to-surface distance between two closed-volume volumetric segmentations (the propagated segmentation and the manual ground truth). In our 4D TEE data, the segmented leaflet envelope does not have a uniform thickness and typically spans approximately from 2 to 4 mm because finite voxel size, partial-volume effects, and resolution differences along axial, lateral, and elevational directions cause 3D echocardiography to depict thin structures as thicker than they truly are [51, 52]; this envelope reflects imaging properties rather than the true anatomical leaflet thickness of 1–1.42 mm from autopsy measurements [43], which enters our framework only as the uniform shell thickness of the FEM medial surface (Fig. 2A) and is not the appropriate reference for the registration metric. The adult 4D TEE values (1.68 ± 0.52 mm for trileaflet and 2.04 ± 0.59 mm for bicuspid) lie within this segmentation envelope. Additionally, a prior inter-observer study of the segmentation method reported a leaflet-averaged mean distance of ~1.05 mm (“Segmentation” section). This value is of the same order as the registration error reported here, indicating that the framework performs close to the variability between expert manual segmentations.

The larger errors observed in 4D CT (4.91 ± 0.83 mm for adult trileaflet and 3.49 ± 1.68 mm for pediatric cases) reflect that the registration pipeline (in particular the voxel-scale-dependent label interpolation reslicing) was tuned for the resolution and noise characteristics of 4D TEE. Even without modality-specific adjustment, the proposed framework reduced the mean distance by 34% on adult CT and 46% on pediatric CT relative to direct registration, demonstrating the impact of the proposed framework on another image modality; the 4D CT results should nonetheless be interpreted as exploratory evidence of generalizability, and modality-specific registration parameter tuning will be required to achieve 4D TEE-level accuracy on CT.

### Physiologically Meaningful Areal Strain Patterns

The following observations for bicuspid and pediatric valves are exploratory given the small subgroup sizes (*n* = 3 for BAV, *n* = 4 for pediatric), and should be interpreted as hypothesis-generating rather than definitive. Areal strain analysis revealed distinct and physiologically meaningful patterns of leaflet deformation across patient groups. Trileaflet adult valves showed positive mean areal expansion across all leaflets, consistent with symmetric valve geometry and normal physiological loading. However, individual leaflet measurements included negative values, indicating contraction even in trileaflet valves. In contrast, bicuspid adult valves exhibited overall leaflet contraction with notable asymmetry: the left and right coronary leaflets showed comparable negative strain, while the non-coronary leaflet displayed smaller contraction. Despite local leaflet expansion up to 20.5%, the overall pattern indicates restricted and uneven leaflet expansion, highlighting the mechanical consequences of fused leaflet geometry and supporting prior reports of asymmetric mechanical loading in bicuspid valves [11-13]. However, inter-leaflet variability in the bicuspid group should be made cautiously due to the limited number of available leaflets, particularly for the non-coronary leaflet. Pediatric valves showed near-zero mean areal strain but notable strain variability, particularly in the right coronary leaflet, likely reflecting ongoing growth and evolving valve geometry. The locations of extreme strain values across groups were consistent with expected deformation patterns as reported previously, with higher strain near the free edge and commissures and lower strain near the annulus [26].

### Green–Lagrange Strain Highlights Local Deformation Differences

Green–Lagrange strain magnitude analysis provided a full measure of local leaflet deformation and complemented the areal strain findings by highlighting differences in deformation across valve groups. Adult trileaflet valves exhibited a relatively uniform mean Green–Lagrange strain magnitude across all three leaflets, suggesting a balanced mechanical loading and relatively symmetric deformation under physiological conditions. Although local variability was present, strain magnitudes were broadly comparable among leaflets, consistent with structurally normal valve. Adult Bicuspid valves showed reduced mean Green–Lagrange strain magnitudes, notably on the non-coronary leaflet, reflecting the asymmetric geometry and altered load sharing associated with leaflet fusion. Although the average strains were lower, bicuspid valves exhibited some of the largest observed maximum strain magnitudes, exceeding 45% on the left coronary leaflet. This combination of reduced mean strain and elevated local maxima highlights the heterogeneous deformation in bicuspid valves, where localized regions may experience substantially higher deformation despite lower overall leaflet averages. This potentially challenges tissue homeostasis and contributes to rapid disease progression. Pediatric valves demonstrated mean Green–Lagrange strain magnitudes comparable to those of trileaflet adult valves, but with greater leaflet-to-leaflet variability and broader strain ranges. In particular, the left coronary leaflet in pediatric valves exhibited strains ranging from 3 to 46%, suggesting significant strain heterogeneity. This increased variability likely reflects developmental differences in tissue structure and leaflet geometry, which may contribute to less uniform mechanical behavior during valve function. The highest Green-Lagrange strain magnitudes were observed along leaflet free edges and near commissure regions, consistent with previous studies reporting strain magnitudes from 17 to 54% [11, 13, 20, 46]. Overall, these findings confirm that trileaflet adult valves exhibit relatively uniform deformation, bicuspid valves show slightly reduced and asymmetric strain patterns, and pediatric valves display more heterogeneous behavior, underscoring the importance of patient-specific mechanical assessment, particularly for valves with asymmetric or developing geometry.

A direct leaflet-specific comparison with the in vivo 3D echocardiography study of Rego et al. [13] is presented in Table 7. Rego et al. report directional surface stretches ($\lambda_{c}$, $\lambda_{r}$), which we converted to Green–Lagrange strain $E_{i}=\left(\lambda_{i}^{2}-1\right)∕2$, magnitude $\|E\|=\sqrt{E_{c}^{2}+E_{r}^{2}}$, and areal strain $\lambda_{c}\lambda_{r}-1$ for consistency with our own measures. Values from the paper text are shown without annotation; values estimated from Fig. 7 of Rego et al. are marked with ≈. Rego et al. confirm that in-plane shear is small (mean shear angle ≈ 10° for all leaflet types), indicating that circumferential and radial stretches dominate the deformation field.

The sign of the areal strain is consistent on a per-leaflet basis: TAV leaflets undergo mean surface expansion in both studies (ours: +5.9 to +7.2%; Rego-derived: +6.1 to +14.0%), whereas the BAV R–N non-fused L leaflet contracts in both studies (ours: −10.6%; Rego-derived: −6.0%). All BAV leaflet types in Rego et al. exhibit circumferential compression ($\lambda_{c}<1$), consistent with the predominantly negative areal strains observed in our BAV group.

The mean Green–Lagrange magnitudes for TAV and BAV leaflets are comparable between the two studies; for example, our TAV L value (17.3%) is approximately 6 percentage points higher than the 11.3% derived from Rego’s TAV L stretches. Three methodological factors account for this offset: (i) our magnitude is computed pointwise from the full-3D strain tensor of the shell element (including bending contributions) and then area-weighted, whereas Rego et al. report purely in-surface stretches averaged over the leaflet and our own verification (Table 5) shows that the in-plane approximation underestimates the full-3D strain by about 7%; (ii) Rego et al. iteratively optimize pressure loading and corrective forces to match the segmented closed-state geometry, whereas our framework applies a uniform 75 mmHg and corrects residual deviations through image registration; and (iii) both studies treat the open reference configuration as strain-free, neglecting residual strains present in vivo. Direct quantitative agreement is therefore not expected, the two pipelines differ substantially in methodology and patient cohort, this comparison constitutes a qualitative cross-study consistency check rather than an independent validation of strain accuracy. The consistent deformation sign and relative magnitude ordering across TAV and BAV leaflets indicate that our strain estimates are physiologically plausible and consistent with prior in vivo reports.

Our peak local Green–Lagrange magnitudes (42.6% for TAV and 45.9% for BAV, both on the left coronary leaflet) are in broad agreement with prior computational studies of aortic valves, which report in-plane strains ranging from 17–32% [11], dynamic FE of congenital BAV at 100 mmHg) to approximately 30–41% [20], FSI of TAV closure at 40–80 mmHg).

### Shear-Dominated Deformation

Effective strain analysis showed the deviatoric (shape-changing) component of leaflet deformation, reflecting changes in shape rather than total deformation. The effective (von Mises-type) strain is computed from the deviatoric part of the total strain tensor, which excludes volumetric contributions, while the Green–Lagrange strain magnitude measures the total local deformation, including volumetric changes. Across all patients and leaflet groups, effective strain values were only slightly lower than the corresponding Green–Lagrange strain magnitudes, indicating that the majority of total deformation is captured by the deviatoric (shear) component rather than by volumetric changes. Mean effective strains were more closely aligned between trileaflet adult and pediatric valves, and the differences between groups were smaller than those observed in the Green–Lagrange strain magnitudes. This exploratory comparison suggests that size- and age-related differences in total deformation are primarily due to volumetric components. In structurally normal trileaflet valves, this indicates that the fundamental shear-dominated deformation mechanisms remain consistent across age groups, despite differences in valve size.

### Limitations and Future Directions

Despite these promising results, several limitations should be acknowledged. The cohort is modest in size (*n* = 20), and the bicuspid (*n* = 3) and pediatric (*n* = 4) subgroups are too small for formal statistical comparison; the corresponding inter-group strain findings are therefore reported as exploratory and require validation in larger, balanced cohorts. The FE model uses a single set of adult-derived isotropic Lee–Sacks parameters and a uniform nominal diastolic pressure (75 mmHg for adults, 45 mmHg for pediatric cases) across the entire cohort, despite known differences in leaflet mechanical properties between pediatric, adult, and pathological (e.g., calcified or fibrotic) tissue. The uncertainty associated with uniform material parameters and uniform nominal pressure has been quantified in the sensitivity analysis (“Effects of FE Modeling Assumptions on Results” and “Impact of FE Modeling Assumptions on Registration Accuracy and Strain”), which bounds the expected range of strain estimates under physiologically plausible parameter variations. However, this bound does not capture differences in collagen fiber architecture, which are not represented in the isotropic formulation, therefore incorporating exact material parameters, patient-specific pressure where available and independent validation of the strain estimates is an important direction for future work, as the sensitivity analysis characterizes the robustness of the strain estimates with respect to modeling assumptions, not their accuracy. Additionally, segmentations were performed manually under expert supervision, introducing operator-dependent variability that was quantified through an inter-observer study for a subset of the adult 4D TEE cohort. Future integration of the proposed framework with 3D deep learning-based segmentation pipelines for cardiac valves [56] could enable fully 4D segmentation, reducing manual effort and improving scalability to larger cohorts. Strain calculations in this study were performed by assuming the open reference aortic valve configuration as the reference, strain-free state and quantifying deformation between the open and closed reference configurations which are extremes of the valve deformation.

Finally, this study focused primarily on proposing the FEM-augmented registration framework and evaluating its performance on limited cases and subgroups as exploratory analysis. Therefore, the direct correlation between clinical outcomes, and geometric and strain analysis will require larger and balanced cohorts to establish relationships between the observed mechanical patterns and clinically relevant measures. Nonetheless, the proposed framework provides an important foundation for such investigations. The detailed strain distributions obtained through the proposed method can provide potential clinical insights: regions of abnormally high or low strain may indicate a higher risk of tissue remodeling, dysfunction, or failure, which could guide repair strategies and risk assessment. Therefore, this framework supports the development of individualized therapeutic strategies and advances our understanding of patient-specific valve behavior, while also providing complementary data to support the training of future learning-based methods.

## Supplementary Material

Supplement

**Supplementary Information** The online version contains supplementary material available at https://doi.org/10.1007/s10439-026-04245-z.

## Figures and Tables

**Table 1:** Finite element model parameters for adult and pediatric aortic valves

Parameter	Adult valves (*n* = 16)	Pediatric valves (*n* = 4)
Constitutive model	Isotropic Lee–Sacks hyperelastic model	
Material parameters	$c_{0}$ = 67 kPa, $c_{1}$ = 13 kPa, $c_{2}$ = 38, k = 5000 kPa, TF = 1	
Element type	Linear triangular shell elements	
Leaflet thickness	1.2 mm	1.0 mm
Solver	Dynamic implicit solver (FEBio)	
Annulus boundary condition	Patient-specific annulus displacement from image registration	
Uniform pressure (aortic side)	75 mmHg	45 mmHg

**Table 2:** Patient characteristics aortic valve

Characteristic	Adult tricuspid (*n* = 13)	Adult bicuspid (*n* = 3)	Pediatric (*n* = 4)
Morphology	Trileaflet	Bicuspid (variable fusion patterns)	Trileaflet (n = 3), Unicuspid (n = 1; partial fusion)
Regurgitation severity	None: 6	None: 1	Moderate: 1
	Mild: 2	Mild: 1	Moderate-to-severe: 1
	Moderate: 2 Moderate-to-severe: 1 Severe: 2	Severe: 1	Severe: 2
Stenosis severity	None: 13	None: 1 Mild: 1 Moderate-to-severe: 1 (calcification)	None: 3 Mild: 1

**Table 3:** Registration accuracy across valve morphologies and imaging modalities

Group	Modality	*n*	Direct registration (mm)	Proposed registration (mm)
Adult trileaflet	4D TEE	11	2.52 ± 0.56	1.68 ± 0.52
Adult bicuspid	4D TEE	3	3.69 ± 0.34	2.04 ± 0.59
Adult trileaflet	4D CT	2	7.47 ± 0.25	4.91 ± 0.83
Pediatric	4D CT	4	6.44 ± 2.32	3.49 ± 1.68
All patients	TEE & CT	20	3.70 ± 2.30	2.23 ± 1.27

Values are mean distance between propagated closed-state segmentation and manual ground truth (mean ± SD)

**Table 4:** Sensitivity analysis: registration accuracy and total strain across 9 parameter configurations for an adult trileaflet case

Run	$c_{1}$ (kPa)	h (mm)	P (mmHg)	GL strain		Areal strain		Mean distance	
				$\varepsilon_{\text{GL}}$	$\Delta \varepsilon_{\text{GL}}\;(\%)$	$\varepsilon_{A}$	$\Delta \varepsilon_{A}\;(\%)$	(mm)	ΔMD(%)
Baseline	13	1.2	75	0.175	—	0.107	—	0.905	—
P low (− 20%)	13	1.2	60	0.159	− 9.1	0.093	− 13.2	0.923	+ 2.0
P high (+20%)	13	1.2	90	0.191	+ 8.8	0.121	+ 13.1	0.917	+ 1.3
h thin (− 17%)	13	1.0	75	0.193	+ 10.3	0.132	+ 23.4	0.892	− 1.4
h thick (+17%)	13	1.4	75	0.161	− 8.4	0.081	− 24.6	0.904	− 0.1
$c_{1}$ soft (− 50%)	6.5	1.2	75	0.194	+ 10.6	0.122	+ 13.7	0.902	− 0.4
$c_{1}$ stiff (+100%)	26	1.2	75	0.162	− 7.6	0.092	− 14.1	0.915	+ 1.2
Corner soft^†^	6.5	1.0	60	0.174	− 1.0	0.101	− 5.8	0.891	− 1.5
Corner stiff	26	1.4	90	0.161	− 8.1	0.079	− 26.1	0.891	− 1.6
Mean ± SD				0.174 ± 0.015		0.103 ± 0.019		0.904 ± 0.012	

The Δ columns show the percentage change from the baseline value.

† Corner soft (Run 8): the FEBio simulation did not converge beyond 43 mmHg (71% of the 60 mmHg target)

**Table 5:** Strain decomposition and in-plane verification across 9 sensitivity analysis runs

Run	Step 1 (FE simulation)				Step 2 (registration)	
	$\varepsilon_{\text{GL},1}^{\text{FEBio}}$	$\varepsilon_{\text{GL},1}^{\text{inp}}\;(\%\text{diff})$	$\varepsilon_{A,1}^{\text{FEBio}}$	$\varepsilon_{A,1}^{\text{geom}}\;(\%\text{diff})$	$\varepsilon_{\text{GL},2}$	$\varepsilon_{A,2}$
Baseline	0.176	0.165 (− 6.7)	0.073	0.073 (0.0)	0.037	0.034
P low	0.160	0.149 (− 7.0)	0.060	0.060 (0.0)	0.036	0.033
P high	0.192	0.179 (− 6.7)	0.090	0.090 (0.0)	0.035	0.032
h thin	0.194	0.186 (− 4.3)	0.099	0.099 (0.0)	0.036	0.033
h thick	0.162	0.150 (− 7.5)	0.048	0.048 (0.0)	0.037	0.033
$c_{1}$ soft	0.195	0.183 (− 5.9)	0.088	0.088 (0.0)	0.036	0.033
$c_{1}$ stiff	0.162	0.147 (− 9.2)	0.058	0.058 (0.0)	0.035	0.034
Corner soft^†^	0.174	0.160 (− 8.4)	0.071	0.071 (0.0)	0.034	0.030
Corner stiff	0.162	0.146 (− 9.8)	0.045	0.045 (0.0)	0.037	0.034
Mean ± SD	0.175 ± 0.015	(−7.3 ± 1.7)	0.070 ± 0.019	(0.0)	0.036 ± 0.001	0.033 ± 0.001

All values are area-weighted means. Step 1: $\varepsilon_{\text{GL},1}^{\text{FEBio}}$, Green–Lagrange magnitude from FEBio (full 3D); $\varepsilon_{\text{GL},1}^{\text{inp}}$, from in-plane geometric calculation; %diff, relative difference vs. FEBio. $\varepsilon_{A,1}^{\text{FEBio}}$, areal strain from FEBio; $\varepsilon_{A,1}^{\text{geom}}$, from geometric calculation. Step 2: $\varepsilon_{\text{GL},2}$, $\varepsilon_{A,2}$, from registration correction.

† Corner soft (Run 8): the FEBio simulation did not converge beyond 43 mmHg (71% of the 60 mmHg target)

**Table 6:** Mean distance (MD) and strain values for representative cases shown in Figs. 4, 5, and 6

Case	Valve type/modality	Direct MD (mm)	Proposed MD (mm)	Mean effective strain (%) (min & max)	Mean areal strain (%) (min & max)
TAV-A	Adult trileaflet/4D TEE	1.96	1.23	11.7 (2.15 & 30.9)	− 3.6 (− 12.2 & 8.0)
TAV-B	Adult trileaflet/4D TEE	2.08	1.27	14.4 (2.91 & 34.2)	3.1 (− 13.4 & 22.1)
TAV-C	Adult trileaflet/4D TEE	2.58	2.43	14.2 (4.6 & 28.1)	− 4.9 (− 19.8 & 9.2)
TAV-D	Adult trileaflet/4D TEE	3.45	2.73	12.2 (2.9 & 36.4)	6.9 (− 12.8 & 26.5)
BAV-A	Adult bicuspid/4D TEE	3.38	1.62	13.2 (4.5 & 41.9)	− 3.2 (− 26.7 & 13.0)
BAV-B	Adult bicuspid/4D TEE	3.63	1.78	14.1 (3.5 & 40.3)	− 10.7 (− 30.4 & 17.6)
BAV-C	Adult bicuspid/4D TEE	4.06	2.71	13.0 (2.6 & 23.4)	− 14.0 (− 26.4 & −1.9)
A	Pediatric trileaflet/4D CT	2.97	1.91	15.5 (4.2 & 34.3)	− 1.8 (− 24.0 & 15.0)
B	Pediatric trileaflet/4D CT	4.50	2.30	15.7 (3.9 & 37.8)	− 0.6 (− 25.5 & 17.6)
C	Pediatric unicuspid/4D CT	9.62	5.12	15.8 (4.5 & 45.0)	2.4 (− 31.1 & 23.3)

Strain values are averaged over all valve leaflets for each case. Maximum and minimum strain values observed on valve’s leaflets are reported in parentheses

**Table 7:** Leaflet-specific comparison in the fully coaptated (closed reference) state between the present study and Rego et al. [13], supplemented by reference strain ranges from computational studies of aortic valves

Group/leaflet	Source	$\lambda_{c}$	$\lambda_{r}$	GL strain ‖E‖ (%)	Areal strain (%)
*Adult trileaflet (TAV)*					
TAV L	Rego 2022	1.034	1.102	11.3	+13.9
TAV R	Rego 2022	1.040	≈ 1.02	≈ 4.6	≈ +6.1
TAV N	Rego 2022	1.056	≈ 1.08	≈ 10.1	≈ +14.0
TAV L	**Present study**	–	–	**17.3**	+**5.9**
TAV R	**Present study**	–	–	**17.6**	+**7.2**
TAV N	**Present study**	–	–	**17.7**	+**6.2**
*Adult bicuspid (BAV)*					
L–R fused cusp	Rego 2022	0.938	≈ 1.15	≈ 17.2	≈ +7.9
L–R non-fused N	Rego 2022	0.916	≈ 1.08	≈ 11.5	≈ −1.1
R–N fused cusp	Rego 2022	0.934	≈ 1.08	≈ 10.5	≈ +0.9
R–N non-fused L	Rego 2022	0.960	0.979	4.4	−6.0
BAV L	**Present study**	–	–	**16.5**	−**10.6**
BAV N	**Present study**	–	–	**11.9**	−**4.6**
BAV R	**Present study**	–	–	**13.8**	−**10.6**
*Reference strain ranges (aortic valves)*					
Jermihov 2011 [11]		TAV & CBAV; FE, 100 mmHg		17–32	–
Marom 2012 [20]		TAV; FSI, 40–80 mmHg		≈ 30– 41	–

Directional surface stretches ($\lambda_{c}$, $\lambda_{r}$); Green–Lagrange (GL) strain $E_{i}=\left(\lambda_{i}^{2}-1\right)∕2$, and GL strain magnitude $\|E\|=\sqrt{E_{c}^{2}+E_{r}^{2}}$; L = left coronary; N = non-coronary; R = right coronary. Present study values are area-weighted means across patients. “–” = not available

## Data Availability

The data will be available upon request to the corresponding authors.
