# Supplementary material for: Biomechanically Informed Image Registration for Patient-Specific Aortic Valve Strain Analysis
Source: Ann Biomed Eng. Author manuscript; Available in PMC 2026 Aug 7. (PMC13450901; doi:10.1007/s10439-026-04245-z)
Supplement: Supplementary file 1 [file NIHMS2199638-supplement-Supplement.docx]

# Proof Corrections

**Manuscript:** *Biomechanically Informed Image Registration for Patient-Specific Aortic Valve Strain Analysis*

**Journal:** Annals of Biomedical Engineering

**Corresponding author:** Mohsen Nakhaei

**Prepared:** 18 June 2026

The following problems were found in the current version. Thank you for your attention.

## 1. Discussion — subsection “Impact of FE Modeling Assumptions on Registration Accuracy and Strain” (first sentence)

**Current:** …the resulting strain estimates (""Effects of FE Modeling Assumptions on Results" section).

**Change to:** …the resulting strain estimates ("Effects of FE Modeling Assumptions on Results" section).

## 2. Figure 2 caption — panel labels (C) and (D) are swapped

**Current:** …(B) Definition of boundary conditions… (D) Estimation of annulus displacement. (C) Imposed uniform diastolic pressure on the leaflets.

**Change to:** …(B) Definition of boundary conditions… (C) Estimation of annulus displacement. (D) Imposed uniform diastolic pressure on the leaflets.

## 3. Please check the size of Figure 7
